# Electroacupuncture Stimulation Regulates Adipose Lipolysis *via* Catecholamine Signaling Mediated by NLRP3 Suppression in Obese Rats

**DOI:** 10.3389/fendo.2021.773127

**Published:** 2022-01-03

**Authors:** Mengjiang Lu, Ziwei Yu, Qian Li, Meirong Gong, Li An, Tiancheng Xu, Mengqian Yuan, Chao Liang, Zhi Yu, Bin Xu

**Affiliations:** ^1^ Key Laboratory of Acupuncture and Medicine Research of Ministry of Education, Nanjing University of Chinese Medicine, Nanjing, China; ^2^ Jiangsu Key Laboratory of Pediatric Respiratory Disease, Institute of Pediatrics, Affiliated Hospital of Nanjing University of Chinese Medicine, Nanjing, China; ^3^ Jiangsu Province Hospital of Chinese Medicine, Affiliated Hospital of Nanjing University of Chinese Medicine, Nanjing, China; ^4^ Medical College, Hebei University of Engineering, Hebei, China

**Keywords:** ES, obesity, nerve-associated macrophage, NLRP3 inflammasome, epididymal visceral adipose tissue

## Abstract

Chronic low-grade inflammation of visceral adipose tissue can cause obesity-associated insulin resistance, leading to metabolic syndrome. However, anti-inflammatory drugs and those for obesity management can lead to serious side effects such as abnormal heart rate and blood pressure. Consequently, this study aimed to explore the therapeutic potential of electroacupuncture stimulation (ES) for obesity and associated chronic inflammation. Sprague-Dawley male rats were fed a high-fat diet (HFD) for ten weeks to build an obesity model, and half of the diet-induced obesity (DIO) rats were received ES. The levels of inflammatory factors were detected by ELISA and qPCR analysis. The nerve-associated macrophages were marked with immunofluorescence staining. The molecular mechanism of NLRP3 inflammasome in ES was determined by the NLRP3 inflammasome activation model. Compared to HDF rats, ES showed decreased body weight and chronic inflammatory damage. Specifically, this occurred *via* a decrease in monoamine oxidase-A (MAOA) expression, which suppressed noradrenaline degradation. MAOA is expressed in nerve-associated macrophages (NAMs), and ES attenuated NAMs by suppressing the NLRP3 inflammasome. The NLRP3 agonist blocked the noradrenaline degradation-reducing effect of ES, and an increase in lipolysis *via* the inhibition of the NLRP3 inflammasome attenuated NAMs. Thus, our findings suggest that ES induced lipolysis *via* activation of the NLRP3 inflammasome in nerve-associated macrophages (NAMs), independently of sympathetic nervous system activity.

## Introduction

Obesity has become a global health issue and poses a substantial social, medical, and economic burden. Obese patients are at a higher risk of mortality due to diabetes or cardiovascular disease-associated events than are normal individuals (1, 2). Furthermore, obesity is associated with a state of chronic, low-grade inflammation in the adipose tissue, which involves adipocyte hypertrophy, macrophage infiltration, and adipocyte macrophage interaction (3). Given this relationship between obesity and inflammation, anti-inflammatory therapy has become a potential new strategy in the clinical management of obesity. It can improve insulin sensitivity and hyperglycemia. For instance, enalapril treatment for 6 months can improve fasting blood glucose levels and increase the ratio of high-molecular-weight adiponectin to total adiponectin in obese patients (4). However, enalapril injection has been reported to cause adverse reactions at the injection site. Moreover, this kind of chronic inflammation requires long-term medication, which leads to safety and financial problems.

Acupuncture is an effective and safe body surface stimulation technique for the treatment of simple obesity. The mechanism of acupuncture associated with weight loss involves adipose lipolysis and thermogenesis (5, 6). Furthermore, electroacupuncture has been reported to induce anti-inflammatory activity in obese rats *via* autonomic nerve effects (7). Our research found that electroacupuncture stimulation (ES) exerts anti-obesity effects *via* inhibition of the norepinephrine transporter protein SlC6a2 in sympathetic-associated macrophages (SAMs) and promotion of thermogenesis through sympathetic nervous system (SNS) activity (8). Thus, the anti-obesity and anti-inflammatory effects of ES may be associated with interactions between the immune and nervous systems.

Despite progress, many challenges and knowledge gaps remain in studying ES. First, although ES can facilitate weight loss and improve chronic low-grade inflammation, the relationship between the anti-inflammatory effect and weight-regulating effects of ES are unclear. Second, the NLRP3 inflammasome has been suggested to affect the regulation of adiposity and insulin sensitivity in the course of obesity, and mice deficient in NLRP3 were reported to be resistant to the development of obesity induced by a high-fat diet (HFD) (9, 10). Activation of the NLRP3 inflammasome upregulates monoamine oxidase-A (MAOA) expression in nerve-associated macrophages (NAMs), which degrade norepinephrine (NE) in visceral adipose tissue (11). ES can suppress the activation of the NLRP3 inflammasome (12, 13). However, it remains unclear whether ES may inhibit NLRP3 in visceral adipose tissue. Nor is it known whether ES can reduce body weight *via* suppression of NLRP3 inflammasome activation. Third, adipose tissues are heterogeneous, in terms of factors such as morphology, function, and immunity (14–16). There are differences in the immune cell population between subcutaneous fat and visceral fat (16). In our previous study, ES activated the sympathetic nerve in inguinal adipose tissue to regulate sympathetic-associated macrophages, reducing norepinephrine transport and increasing fat thermogenesis (8). However, it is unclear whether ES can mediate immunity in visceral adipose and the related mechanism. As such, it remains to be determined whether ES-attenuated NLRP3 inflammasome activation in visceral adipose could suppress MAOA expression in NAMs and induce lipolysis in visceral adipose tissue. In this study, we evaluated the relationship between obesity and inflammation *via* the application of ES. We hypothesized that the anti-inflammatory and weight loss-inducing mechanisms of ES are associated with interactions between the immune and nervous systems.

## Material and Methods

### Animals

Weaned (3-week-old) Sprague-Dawley male rats (Model Animal Research Center of Nanjing Medical University, China) were divided into the normal diet (ND) and HFD groups. All animals were housed under controlled environmental conditions (22°C, 40-60% relative humidity, 12-/12-h light/dark cycle) and were given free access to water and food. All animals were allowed 1 week of feeding adaptation. The ND group was given a regular diet, and the HFD group was given a diet comprising 45 kcal% fat (Research Diets, D12451). After feeding for 10 weeks, part of the HFD group served as a control group, while others received ES and drug treatment. To maintain the state of obesity, the animals were fed the high-fat diet until the end of the experiment. All experiments were performed according to the Principles of Laboratory Animal Care and the Guide for the Care and Use of Laboratory Animals published by the National Science Council, China (Animal Committee Number: 201809A015).

### ES

The animals were anesthetized with isoflurane (0.5%-1.5%) for ES. Electroacupuncture was performed at the forelimb LI11 (Quchi) acupoint by inserting a pair of stainless-steel acupuncture needles (diameter: 0.3 mm) up to 3 mm in depth at each site. LI11 is located midway between the lateral end of the transverse cubital crease and the lateral humeral epicondyle (17). The needles were connected to Han’s electroacupuncture instrument (LH402A; Beijing Huawei Technologies Co. Ltd). ES was performed with a stimulation current of 2 mA, a pulse width of 0.4 ms, an alternating frequency between 2 and 15 Hz every 3 s, and a stimulation time of 20 min. The procedure was performed six times a week and lasted for 4 weeks.

### Treatment With an NLRP3 Inflammasome Agonist

The rats from the HFD group were administered the NLRP3 inflammasome agonist (nigericin sodium salt) and were assigned to three groups: (1) solvent control (SC); (2) nigericin sodium salt treatment (Nig); (3) nigericin sodium salt with ES (Nig+ES) groups. After 10 weeks of HFD feeding, the solvent control and Nigericin sodium salt (M7029, AbMole, USA) were administered in the epididymal visceral adipose tissue (eWAT) in rats *via* intraperitoneal injection (i.p.) before ES. The NLRP3 agonist (Nig) was administered three times a week (Nig 1 mg/kg body weight) for 4 weeks.

### Hematoxylin and Eosin Staining of White Adipose Tissue

The adipose tissues were fixed in 4% paraformaldehyde and embedded in paraffin, sectioned at 8-μm thickness. Hematoxylin and eosin staining (H&E staining) were performed according to the standard process. We used Image-Pro Plus software to collect image fields of each rat for adipocyte area, and more than 250 cells per rat were selected for measuring (18).

### Western Blot

eWAT was harvested using the Adipose Protein Extraction Kit (MinuteTM) for protein extraction. Eighty milligrams of adipose tissue was homogenized with 250 ml of extraction buffer. After centrifugation at 2,000 g for 1 min, the tissue was transferred to a filter cartridge with a collection tube and incubated at -20°C for 20 min. After incubation, the tissue was immediately centrifuged at 2000 rpm for 1 min with the cap open. The flow-through contained total proteins from the adipose tissue. Then, 30 mg of total tissue lysate was separated by SDS-PAGE (10% separation gel and 5% concentration gel). Electrophoresis was performed at 70 V for 0.5 h and 110 V for 1 h. The protein bands were transferred to a PVDF membrane using e-Blot (Genscript), and 5% BSA was added for 1 h to block the membrane. The membrane was then made to react with primary antibodies against ATGL (1:1000, Abcam), HSL (1:3000, Abcam), p-HSL (1:1000, Sab), NLRP3 (1:1000, Abcam), Caspase-1 (1:1000, Abcam), IL-1β (1:1000, Abcam), MAOA (1:1000, Abcam), β-actin (1:1000, Abcam), and tyrosine hydroxylase (1:1000, Abcam) at 4°C overnight. Incubation with the corresponding secondary antibodies (diluted to 1:3000, Abcam) was performed at room temperature (18°C -25°C) for 1 h.

### Immunohistochemistry

After tissue perfusion and separation, the samples were fixed with 4% paraformaldehyde at 4°C over 12 h and then immersed in 30% sucrose solution at 4°C overnight. Tissues were embedded in optimal cutting temperature (OCT) compounds, and sections (thickness, 50 μm) were prepared using a freezing microtome (Leica, German). The sections were blocked with 0.3% Triton X-100 Sea Block blocking buffer (Thermo Fisher Scientific, United States) for 1 h, and incubated with primary antibodies against the following proteins at 4°C overnight: tyrosine hydroxylase (TH, 1:500, Abcam) and F4/80 (1:50, Sab). After washing with PBS three times, the sections were incubated with secondary antibodies: anti-Alexa Fluor 488, Alexa Fluor 594 goat anti-chicken, and anti-rabbit (1:500, Abcam) antibodies at room temperature for 1 h. For samples were mounted using the ProLong Diamond Antifade Mountant (Thermo Fisher Scientific, United States) and cured for 24 h.

### RT-PCR

Total RNA was extracted from 300 mg of eWAT by using Trizol (TaKaRa, Japan). The total RNA was reverse-transcribed to produce cDNA by using a thermal cycler (Bio-Rad, United States) with PrimeScript RT Master Mix (TaKaRa, Japan) at 37°C for 15 min and 98°C for 15 s. The PCR reaction mixture (20 mL) contained 4 μL of a forward primer, 4 μL of a reverse primer, 2 μL of cDNA, and 10 μL of SYBR Green Mix (TaKaRa, Japan). The PCR protocol was as follows: 40 cycles of amplification for 30 s at 95°C, 5 s at 95°C, and 30 s at 60°C. Data were processed using the 2^-△△CT^ method. The primers used in the experiment are shown in **Table 1**.

**Table 1:** Sequences of the primers used for real-time PCR.

Gene names	Primer Sequence	
GAPDH	Forward	5′-GGT GCT GAG TAT GTC GTG GAG-3′
	Reverse	5′-GTC TTC TGA GTG GCA GTG ATG-3′
IL-1b	Forward	5′-CCT CGT GCT GTC TGA CCC AT-3′
	Reverse	5′-CAA ACC GCT TTT CCA TCT TCT TC-3′
TNFα	Forward	5′-TGC CTC AGC CTC TTC TCA TTC C-3′
	Reverse	5′-TCC TCC GCT TGG TGG TTT G-3′
Il-10	Forward	5′-CCA GTC AGC CAG ACC CAC AT-3′
	Reverse	5′-AAT CAT TCT TCA CCT GCT CCA C-3′

### Detection of Serum Free Fatty Acid, Cholesterol, and Triglyceride Level

Serum level were detected according to the manufacturer’s recommendations. For free fatty acid, cholesterol and triglyceride, standards and each 2.5 μl of mice serum was added into 96 well coated plate with 250μl Working fluid, incubated at 37°C for 10 min. OD values were detected at 510 nm by using microplate reader (Bio-Tek, USA). Free fatty acid, cholesterol and triglyceride level were quantified by standard curve.

### ELISA

Plasma concentrations of TNFα, IL-6,IL-4, IL-1β, IL-18 were assayed by ELISA. Standard or sample solution (100 mL) was added to 96-well polystyrene microplates and incubated at 37°C for 90 min. Then, 10 mL of biotinylated antibodies (Nanjing Jiancheng Bioengineering Institute, Nanjing) was added to the wells and incubation was performed at 37°C for 60 min. The solution in each well was then aspirated, and each well was washed three times with 350 mL of 1* wash buffer. Next, 100 mL of tetramethylbenzidine (TMB) substrate was added to each well and incubation was performed for 10 min. To stop the reaction, 100 mL of stop solution was added, and the absorbance in the wells was read at 450 nm.

### Caspase-1 Activity Assay

The caspase-1 activity in eWAT was measured by Abbkine Caspase 1 Assay Kit (Colorimetric). This assay is performed on the basis of the ability of caspase-1 to change acetyl-Tyr-Val-Ala-Asp p-nitroaniline (Ac-YVAD-pNA) into the yellow formazan product pNA. After treatment, the adipose tissues were harvested and lysed. The protocol was determined according to the manufacturer’s instructions. Absorbance was measured at 405 nm by microplate reader (Bio-Tek, USA). Standard curves for the assay system were obtained from dilutions of the standards of pNA. Caspase-1 activity was then obtained by determining the amount of pNA according to the standard curve of pNA.

### Statistical Analysis

GraphPad Prism6 was used for statistical analysis. Data are expressed as mean ± SEM values. To test whether obtained data were normally distributed the Shapiro-Wilk test was performed. Levene’s test was used to assess homogeneity of variance. Data between two groups were analysed by unpaired t-test, if the data were in Gaussian distribution and had equal variance, or by Mann-Whitney rank-sum test if the data were not normally distributed. Data among more than two groups were compared using one-way analysis of variance (ANOVA) followed by *post hoc* Student and Newman-Keuls tests. P < 0.05 was considered to indicate statistical significance.

## Results

### ES Reduced Body Weight and Systemic Inflammation in the HFD Group

**Figure 1:**
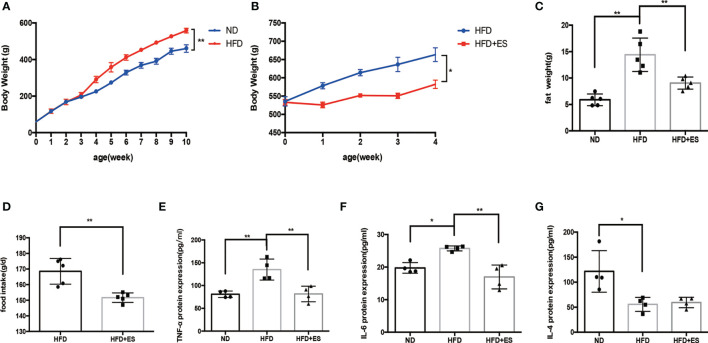
Effect of ES on body weight, adipose weight, and inflammatory state. **(A)** Bodyweight of rats from the normal diet (ND) and high-fat diet (HFD) groups after 10 weeks (n = 5, ^**^ *P <* 0.01). **(B)** Bodyweight of HFD rats with or without ES during 4 weeks after feeding for 10 weeks (n = 5, ^*^ *P <* 0.05). **(C)** The weight of eWAT in the ND, HFD, and HFD + ES groups (n = 5, ^**^ *P <* 0.01). **(D)** Food intake of HFD and HFD+ES mice. Data are shown as average food intake per day from 4 weeks of ES (n = 5, ^**^ *P <* 0.01). **(E)** Relative TNF-α expression in serum (n = 4, ^**^ *P <* 0.01). **(F)** Relative IL-6 expression in serum (n = 4, ^*^ *P <* 0.05, ^**^ *P <* 0.01). **(G)** Relative IL-4 protein expression in serum (n = 4, ^*^ *P <* 0.05).

We first compared the bodyweight of the ND and HFD groups. HFD feeding for 10 weeks induced obesity (**Figure 1A**). eWATs were weighed after 10 weeks of HFD feeding (**Figure 1C**). Since HFD consumption not only leads to obesity but also leads to low-grade inflammation (19), we measured blood cytokine levels. The proinflammatory cytokine IL-6 and TNF-α significantly accumulated in blood serum in the HFD group (**Figures 1E, F**), while the level of the anti-inflammatory cytokine IL-4 reduced in this group (**Figure 1G**). ES at ST25 reduced body weight (20), and eWAT analysis showed that ES at LI11 (2.0 mA) reduced body weight and fat weight (**Figures 1B, C**). Also, ES reduced food intake during 4 weeks (**Figure 1D**). Interestingly, ES at LI11 reduced blood serum levels of the proinflammatory cytokine IL-6 and TNF-α in the HFD group (**Figures 1E, F**) but did not promote the expression of anti-inflammatory cytokines, such as IL-4 (**Figure 1G**).

### ES Attenuated Adipose Inflammation and Dyslipidemia

**Figure 2:**
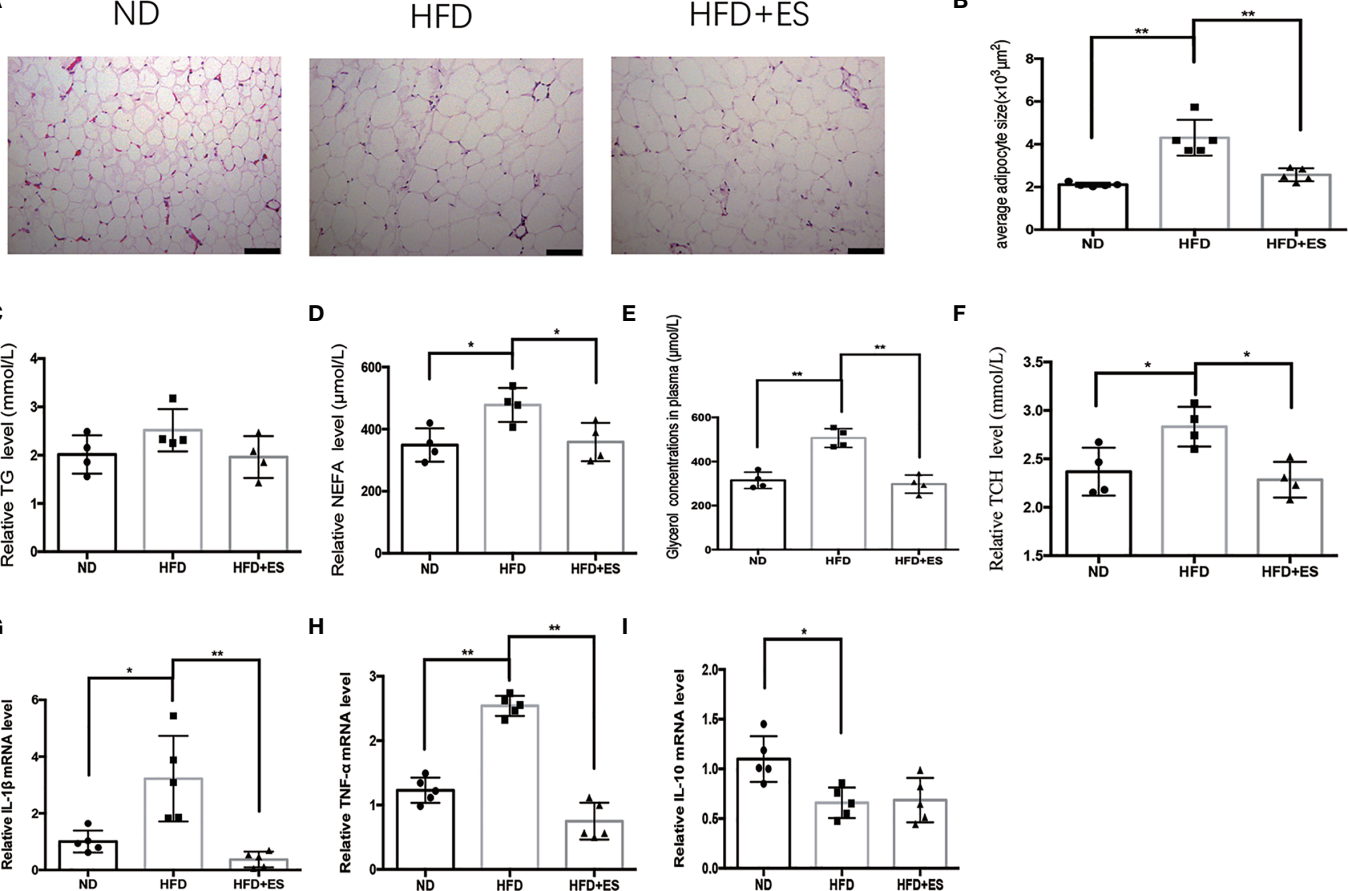
ES attenuated adipose inflammation and dyslipidemia. **(A)** Representative hematoxylin and eosin (H&E) staining sections (scale bar, 100 μm). **(B)** Adipocyte size in eWAT from the ND, HFD, and HFD+ES groups was measured (n = 5, ^**^ *P*<0.01). **(C)** Serum levels of triglyceride (TG) in the ND, HFD, and HFD+ES groups (n = 4). **(D)** Serum levels of non-esterified fatty acid (NEFA) in the ND, HFD, and HFD+ES groups (n = 4, ^*^ *P <* 0.05). **(E)** Serum levels of glycerol in the ND, HFD, and HFD+ES groups (n = 4, ^**^ *P <* 0.01). **(F)** Serum levels of total cholesterol (TCH) in the ND, HFD, and HFD + ES groups (n = 4, ^*^ *P <.*05). **(G)** Relative IL-1β mRNA expression in eWAT (n = 5, ^*^ *P <* 0.05, ^**^ *P <* 0.01). **(H)** Relative TNF-α mRNA expression in eWAT (n = 5, ^**^ *P <* 0.01). **(I)** Relative IL-10 mRNA expression in eWAT (n = 5, ^*^ *P <* 0.05).

Obesity is associated with dynamic adipose tissue remodeling characterized by adipocyte hypertrophy, especially in the visceral adipose tissue (21, 22). The size of adipocytes in eWAT increased in the HFD group and reduced on ES (**Figures 2A, B**). Visceral fat is the largest lipid reservoir in the body, and its excessive expansion causes lipid metabolism disorder (23). As shown in **Figures 2C–F**, HFD induced dyslipidemia, including abnormal triglyceride (TG), non-esterified fatty acid (NEFA), and total cholesterol (TCH) levels. ES significantly reduced NEFA and TCH levels. As the low-grade inflammation caused by visceral adipose tissue inflammatory accumulate, we measured cytokine levels in eWAT. As shown in **Figures 2G–I**, the mRNA expression of the proinflammatory cytokine IL-1β and TNF-α in eWAT significantly increased in the HFD group compared to that in the ND group and decreased on ES compared to that in the HFD group. Thus, there may be a connection between lipid metabolism and inflammation in ES.

### ES Induced Lipolysis by Attenuating the Activation of MAOA in eWAT

**Figure 3:**
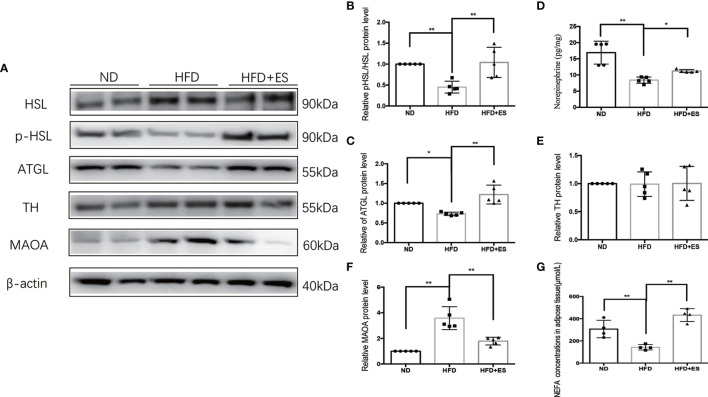
ES induced lipolysis by attenuating the activation of MAOA in eWAT. **(A)** Immunoblot image showing ATGL, HSL, p-HSL, TH, and MAOA in the ND, HFD, and HFD+ES groups. **(B)** Relative p-HSL/HSL protein expression in eWAT (n = 5, ^**^ *P <* 0.01). **(C)** Relative ATGL protein expression in eWAT (n = 5, ^*^ *P <* 0.05, ^**^ *P <* 0.01). **(D)** Relative norepinephrine expression in eWAT (n = 5, ^*^ *P <* 0.05, ^**^ *P <* 0.01). **(E)** Relative TH protein expression in eWAT (n = 5). **(F)** Relative MAOA protein expression in eWAT (n = 5, ^**^ *P <* 0.01). **(G)** NEFA concentrations in Ewat (n = 4, ^**^ *P <* 0.01).

As shown in **Figures 3A–C, G**, ES induced lipolysis by activating p-HSL and ATGL. The SNS plays a crucial role in facilitating noradrenaline (NE) to stimulate lipolysis in white adipose tissue (WAT) (24). Next, we measured the level of NE in eWAT. The level of NE was higher in ES than that in the HFD group (**Figure 3D**). Consequently, we examined TH expression, which is related to NE generation (**Figures 3A, E**). The TH level in ES was not significantly greater than that in the HFD mice. Thus, NE generation did not increase, and we considered that ES might attenuate the degradation of NE. MAOA was an essential enzyme in the metabolism of monoamines, which can degrade NE in adipose tissue (25). As shown in **Figures 3A, F**, ES attenuated the activation of MAOA in eWAT. The results indicated that ES induced lipolysis by attenuating the activation of MAOA in eWAT but did not increase NE generation.

### ES Suppression of NAMs in eWAT

**Figure 4:**
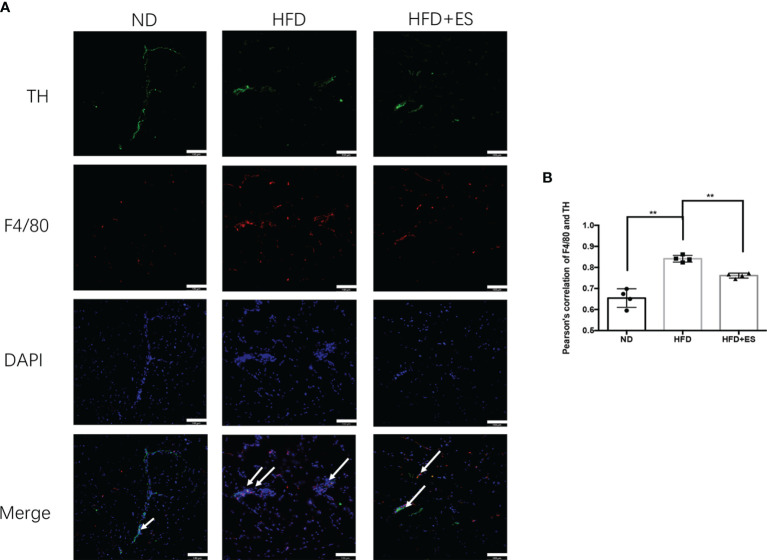
ES suppression of nerve-associated macrophages (NAMs) in eWAT. **(A)** Representative images showing TH (green), F4/80 (red), and DAPI (blue) fluorescence (scale bar, 100 μm). The white arrows in the merged image indicate NAMs. **(B)** Correlation between F4/80 and TH as indicated by Pearson correlation analysis (n = 4, ^**^ *P* < 0.01).

The presence of NAMs has been recently reported in adipose depots. In inguinal subcutaneous white adipose tissue (iWAT), inflammatory NAMs express machinery for the degradation (MAOA) of noradrenaline, thereby dampening β3-adrenergic receptor (β3-AR)-driven lipolysis (26). In obesity, NAMs accumulate in eWAT (**Figure 4A**). As shown in **Figure 4B**, ES decreased the co-localization expression of F4/80 (macrophage) and TH (sympathetic nerve), which were labeled NAMs. These findings indicate that ES may induce lipolysis through immune adjustment.

### ES Regulated NAM *via* Suppression of the NLRP3 Inflammasome

**Figure 5:**
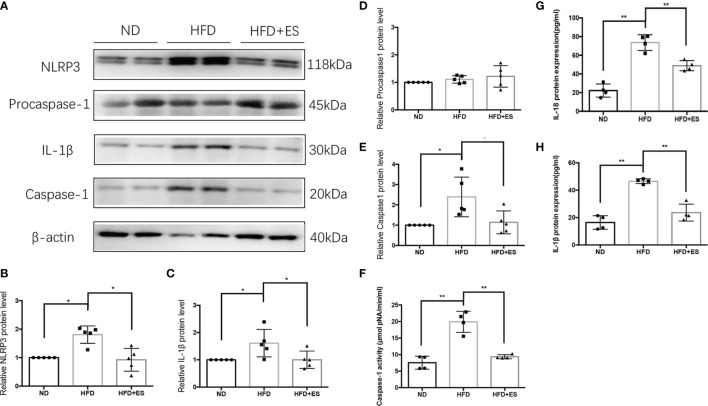
ES regulated NAM *via* suppression of the NLRP3 inflammasome. **(A)** Immunoblot image showing NLRP3, Procaspase-1, caspase-1, and IL-1β in the ND, HFD, and HFD+ES groups. **(B)** Relative NLRP3 protein expression in eWAT (n = 5, ^*^ *P <* 0.05). **(C)** Relative IL-1β protein expression in eWAT (n = 5, ^*^ *P <* 0.05). **(D)** Relative Procaspase-1 protein expression in eWAT (n = 5). **(E)** Relative caspase-1 protein expression in eWAT (n = 5, ^*^ *P <* 0.05). **(F)** Relative caspase-1 activity in eWAT (n = 4, ^*^ *P <* 0.05). **(G)** Relative IL-18 expression in serum (n = 4, ^**^ *P <* 0.01). **(H)** Relative IL-1β expression in serum (n = 4, ^**^ *P <* 0.01).

**Figure 6:**
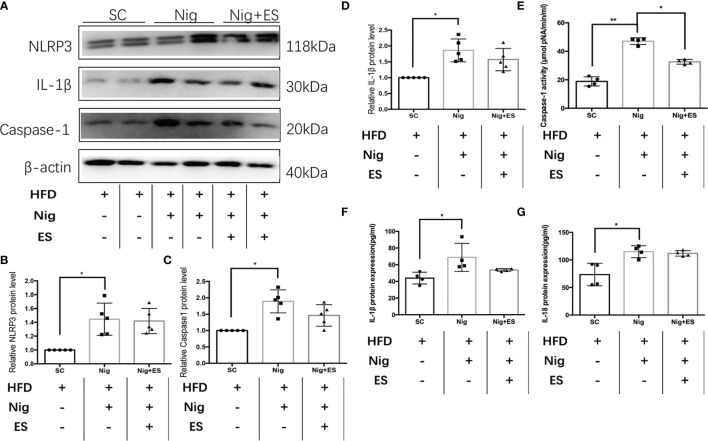
The NLRP3 inflammasome agonist activated the NLRP3 inflammasome in the ES group. **(A)** Immunoblot image showing NLRP3, caspase-1, and IL-1β in the SC group, Nig group, and Nig+ES group. **(B)** Relative NLRP3 protein expression in eWAT (n = 5, ^*^ *P <* 0.05). **(C)** Relative caspase-1 protein expression in eWAT (n = 5, ^*^ *P <* 0.05). **(D)** Relative IL-1β protein expression in eWAT (n = 5, ^*^ *P <* 0.05). **(E)** Relative caspase-1 activity in eWAT (n = 4, ^**^ *P <* 0.01, ^*^ *P <* 0.05). **(F)** Relative IL-18 expression in serum (n = 4, ^*^ *P <* 0.05). **(G)** Relative IL-1β expression in serum (n = 4, ^*^ *P <* 0.05).

**Figure 7:**
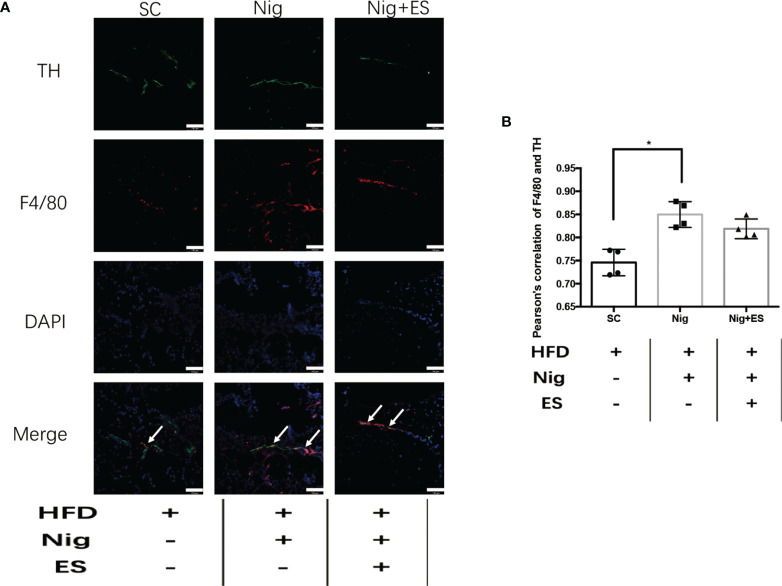
NLRP3 inflammasome agonist induced the NAMs in the ES group. **(A)** Representative images showing TH (green), F4/80 (red), and DAPI (blue) fluorescence in the SC, Nig, and Nig+ES groups(scale bar, 100 μm). The white arrows in the merged image indicate NAMs. **(B)** Correlation between F4/80 and TH as indicated by Pearson correlation analysis (n = 4, ^*^ *P* < 0.05).

In eWAT, the increasing activity of the NLRP3 inflammasome leads to increases in MAOA oxidation, thereby dampening lipolysis and promoting obesity (11). Therefore, we hypothesized that ES might attenuate the activation of MAOA in NAMs by suppressing the activation of the NLRP3 inflammasome. As shown in **Figures 5A–E**, ES attenuated the relative protein level of the NLRP3 inflammasome, including NLRP3, caspase-1, and IL-1β, but not procaspase-1. In **Figure 5F**, ES attenuated the activity of caspase-1. Also, we measured the cytokines processed by the NLRP3 inflammasome such as IL-1β and IL-18. ES decreased the level of IL-1β and IL-18 in the serum (**Figures 5G, H**). To verify whether ES regulated NAM *via* suppression of the NLRP3 inflammasome, we injected the NLRP3 inflammasome agonists (Nigericin sodium salt) into the abdominal cavity of rats. As shown in **Figures 6A–E**, the agonist activated the NLRP3 inflammasome in the Nig and Nig+ES groups, which increased the expression of NLRP3, caspase-1, IL-1β and the activity of caspase-1 in eWAT. Also, the agonist increased the production of IL-1β and IL-18 in the serum (**Figures 6F, G**). It also increased the co-localization expression of F4/80 (macrophage) and TH (sympathetic nerve), which blocked the effect of ES-regulated NAMs (**Figures 7A, B**). These data suggest that ES regulated NAM *via* suppression of the NLRP3 inflammasome.

### NLRP3 Inflammasome Agonist Suppressed ES-Mediated Lipolysis

**Figure 8:**
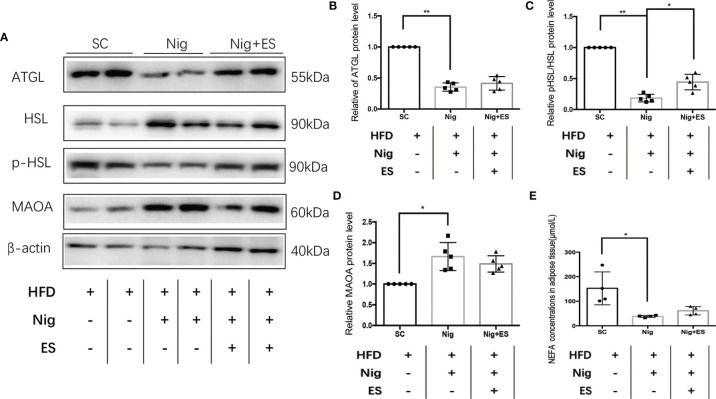
NLRP3 inflammasome agonist suppressed ES-mediated lipolysis. **(A)** Immunoblot image showing ATGL, HSL, p-HSL, and MAOA in the SC, Nig, and Nig+ES groups. **(B)** Relative ATGL protein expression in eWAT (n = 5, ^**^ *P <* 0.01). **(C)** Relative p-HSL/HSL protein expression in eWAT (n = 5, ^**^ *P <* 0.01, ^*^ *P <* 0.05). **(D)** Relative MAOA protein expression in eWAT (n = 5, ^*^ *P <* 0.05). **(E)** NEFA concentrations in eWAT (SC, Nig, and Nig+ES groups, n = 4, ^*^ *P <* 0.05).

To verify whether the NLRP3 inflammasome agonist inhibited ES-induced lipolysis, we tested the enzymes related to lipolysis. As shown in **Figures 8A, D**, the agonist increased the expression of MAOA oxidation, suggesting that the catecholamine signal pathway was inactivated. Furthermore, it decreased the expression of p-HSL/HSL and ATGL. Thus, ES suppressed the NLRP3 inflammasome to attenuate the activation of MAOA, which induces lipolysis in eWAT (**Figures 8A–C, E**).

## Discussion

This is the first study to clarify that ES can reduce the expression of MAOA and the degradation of NE by inhibiting NLRP3-mediated NAMs to induce visceral adipose lipolysis. The process of obesity is accompanied by an increase in fat synthesis, especially visceral fat. Furthermore, the accumulation of adipose tissue leads to recruitment of macrophages, which releases proinflammatory cytokines (3). Previous studies showed that ES reduces body weight, down-regulates the level of TNF-α, and up-regulates the serum levels of IL-10 in HFD rats (27, 28). In our current study, we characterized the effects of ES in reducing body weight and the chronic inflammatory state. We found that ES LI11 reduced body weight and proinflammatory cytokines like IL-6 and TNF-α, but did not promote anti-inflammatory cytokines like IL-4. A previous study indicated that ES improved the adipose tissue inflammatory response in obese rats, at least partly *via* attenuation of lipogenesis signaling (29). In our study, ES was found to increase the lipolysis in eWAT, in addition to the expression of HSL and ATGL. Interesting, ES decrease the serum level of NEFA. Although adipose lipolysis should increase NEFA, reduced food intake and increased energy consumption will reduce NEFA (30). Our results showed that ES reduced food intake. Previous studies also showed that electroacupuncture increased WAT browning (increased energy consumption) (31), consistent with our results that ES did not increase NEFA. On the other hand, the NEFA in weight loss is a dynamic change that rises first then decreases (32). ES was also shown to reduce the level of proinflammatory mediators in eWAT.

Obesity is associated with a reduction in catecholamine-stimulated lipolysis (33). A previous study showed that the activation of WAT SNS innervation is the primary initiator of lipolysis (34). SNS innervation facilitated by noradrenaline release drives β3-adrenergic receptors (β3-AR) in white and brown adipocytes to stimulate lipolysis. In the present study, ES was shown to promote the protein and mRNA expressions of UCP1, PRDM16, and PGC-1α in adipose tissue, leading to thermogenesis and increasing energy consumption, and it activated sympathetic nerves *via* p-TH, A2AR, and β3AR in WAT (35). In our study, ES LI11 increased the level of NE in eWAT but did not activate sympathetic nerves *via* TH.

Biosynthesis and the metabolism of serotonin and catecholamines involve at least eight individual enzymes mainly expressed in tissues derived from the neuroectoderm, such as sympathetic nerves. The main biosynthetic enzyme is tyrosine hydroxylase (TH), and the specific catabolic enzyme is MAOA. The classic theory suggests that adrenergic neurons primarily use the A form of MAO to regulate endogenous catecholamine stores and that MAOA levels responded to these neurons’ physiologic activity (36, 37). Our research showed that ES suppresses MAOA in obesity, which decreased the degradation of NE. This is the first study to show that ES may increase lipolysis from catecholamine signals but independent sympathetic activation.

Two noradrenaline-degrading macrophage populations in adipose tissue have been recently identified simultaneously and were landmark discoveries that demonstrated the direct immunomodulatory role of immune cells in the sympathetic innervation of adipocytes. In subcutaneous WAT, the sympathetic neuron-associated macrophages (SAMs) exhibit enriched expression of the noradrenaline transporter SLC6A2 and degradation enzyme MAOA. SAMs are increased in obese mice, and mice that lack Slc6a2 from SAMs significantly inhibit NE uptake by SAMs. This process enhances sympathetic signaling to the adipose tissue and improves energy homeostasis (26). In our previous study, ES ST25 decreased the expression of Slc6a2 from SAMs in iWAT but not in eWAT (8). A SAM-like macrophage population that also degrades noradrenaline was simultaneously identified in visceral WAT by Camell and colleagues (11). For these macrophages, MAOA upregulation led to noradrenaline degradation and decreased lipolysis in aged mice. In our study, the co-localization correlation coefficient of F4/80 and TH, which represent neuro-associated macrophages (NAMs), increased in obese rats. Furthermore, ES LI11 decreased the Pearson’s correlation of TH and F4/80, which labeled NAMs.

Although the two types of macrophages have similar functions, the heterogeneity of different adipose tissues leads to different immune cell subsets. In visceral WAT, the NAM is inflammasome-dependent. The activation of NLRP3 increased the expression of MAOA in NAMs, resulting in the degradation of catecholamines like NE, and thereby impairing lipolysis (11). The NLRP3 inflammasome complex is an intracellular platform that can sense and respond to a wide range of pathogen-associated molecular patterns (PAMPs) and danger-associated molecular patterns (DAMPs). NLRP3 inflammasome assembly and activation of caspase-1 can cleave pro-IL-1β into the bioactive forms, which are then available to be secreted out of the cell. The secreted IL-1β can enter the circulation or local extracellular environment and bind and signal through the IL-1R (IL-1 receptor) (38). Obesity-associated PAMPs and DAMPs can activate the NLRP3 inflammasome. Here, we found a high NLRP3 expression in obese rats, and caspase-1 and IL-1β expression also increased. Our colleagues found that electroacupuncture pretreatment (EAP) inhibits the activation of the NLRP3 inflammasome, promotes M2 polarization of macrophages, and reduces the recruitment of neutrophils in damaged myocardium (39). In our study, ES LI11 suppressed the activation of the NLRP3 inflammasome and the expression of caspase-1 and IL-1β.

This is the first study to show an immune-dependent way for ES to reduce body weight and promote lipolysis. ES LI11 decreased MAOA expression *via* suppression of the NLRP3 inflammasome from eWAT. The NLRP3 inflammasome agonist inhibited ES to suppress MAOa in eWAT. Furthermore, activation of the NLRP3 inflammasome also attenuated ES-suppressed NAMs, which increased the degradation of NE and decreased adipose lipolysis.

## Conclusion

In conclusion, we showed ES LI11 induced lipolysis *via* catecholamine signals but with independent sympathetic activation. ES LI11 increased lipolysis *via* inhibiting the activation of NLRP3 inflammasome.

## Data Availability Statement

The original contributions presented in the study are included in the article/**Supplementary Material**. Further inquiries can be directed to the corresponding authors.

## Ethics Statement

The animal study was reviewed and approved by the Experimental Animal Ethics Committee of Nanjing University of Chinese Medicine.

## Author Contributions

ML and ZWY contributed equally to this work. BX, ZY, and ML conceived and designed the experiments. ML and ZWY performed the experiments and wrote the manuscript. QL, MG, LA, TX, MY, and CL performed the experiments. QL and ZY analyzed the data. All authors read and approved the final version of article to be published.

## Funding

This work was supported by the National Natural Science Foundation of China (Nos. 81873238, 82074532, 81904290), the Natural Science Foundation of the Higher Education Institutions of Jiangsu Province, China (Grant No. 21KJB360001), the Priority Academic Program Development of Jiangsu Higher Education Institutions (PAPD), the Open Projects of the Discipline of Chinese Medicine of Nanjing University of Chinese Medicine Supported by the Subject of Academic Priority Discipline of Jiangsu Higher Education Institutions (No. ZYX03KF012), the Leading Talents of Traditional Chinese Medicine in Jiangsu (No. SLJ0225), and the Natural Science Foundation of Hebei Province (No. H2019402360).

## Conflict of Interest

The authors declare that the research was conducted in the absence of any commercial or financial relationships that could be construed as a potential conflict of interest.

## Publisher’s Note

All claims expressed in this article are solely those of the authors and do not necessarily represent those of their affiliated organizations, or those of the publisher, the editors and the reviewers. Any product that may be evaluated in this article, or claim that may be made by its manufacturer, is not guaranteed or endorsed by the publisher.
