# Supplementary figures and images for: Electroacupuncture Stimulation Regulates Adipose Lipolysis via Catecholamine Signaling Mediated by NLRP3 Suppression in Obese Rats
Source: Front Endocrinol (Lausanne). 2022 Jan 3;12:773127. doi: 10.3389/fendo.2021.773127 (PMC8762326; doi:10.3389/fendo.2021.773127)

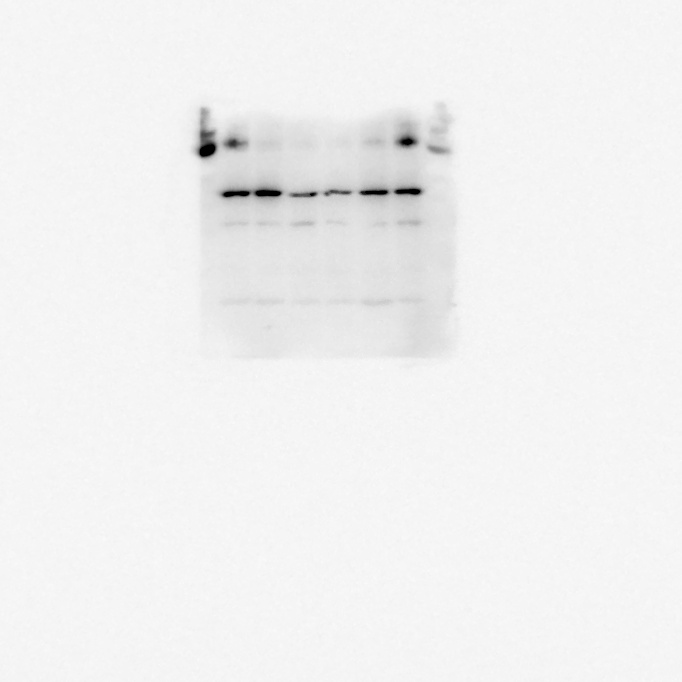

Supplement: Supplementary file 1 [file DataSheet_1.zip › original WB images/ATGL-nig.tiff]

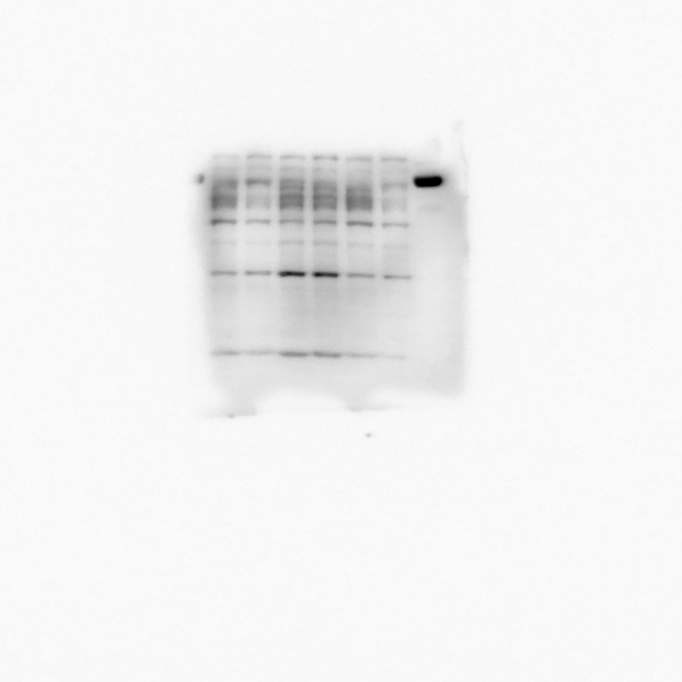

Supplement: Supplementary file 1 [file DataSheet_1.zip › original WB images/casepase1-ES.tiff]

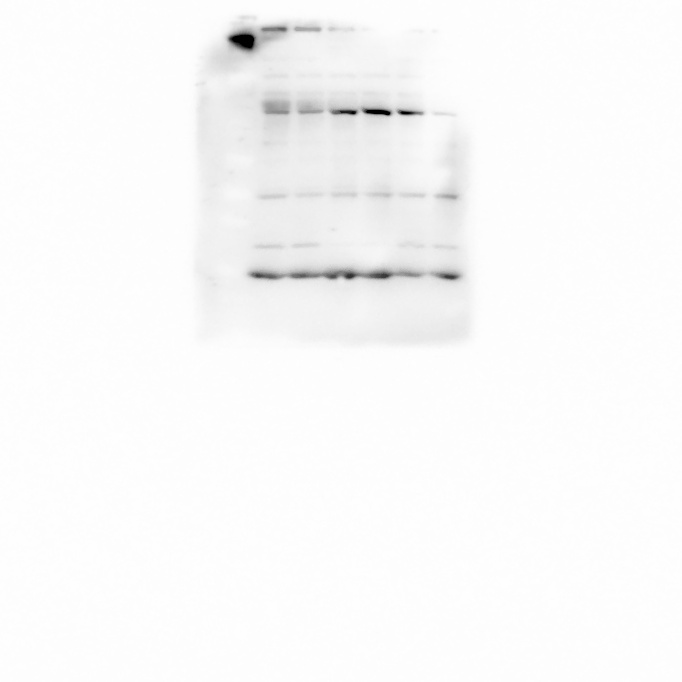

Supplement: Supplementary file 1 [file DataSheet_1.zip › original WB images/MAO-Es.tiff]

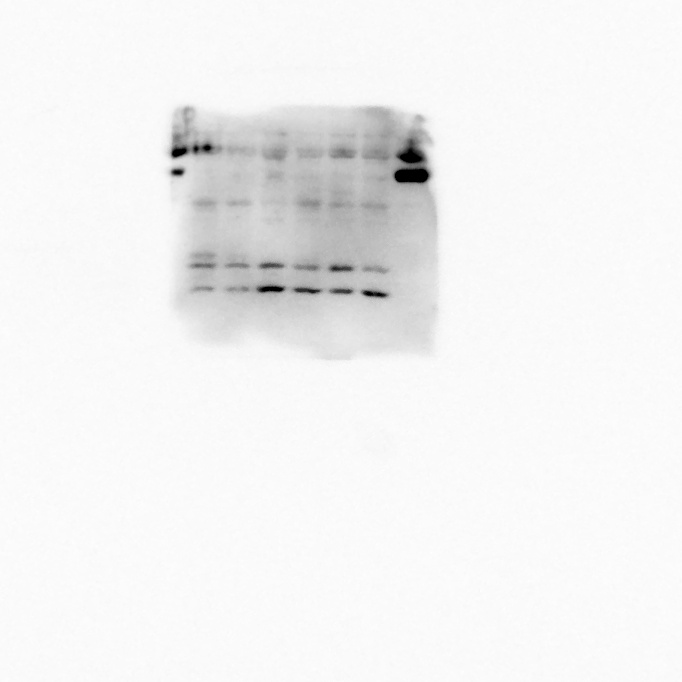

Supplement: Supplementary file 1 [file DataSheet_1.zip › original WB images/IL-1beta-nig.tiff]

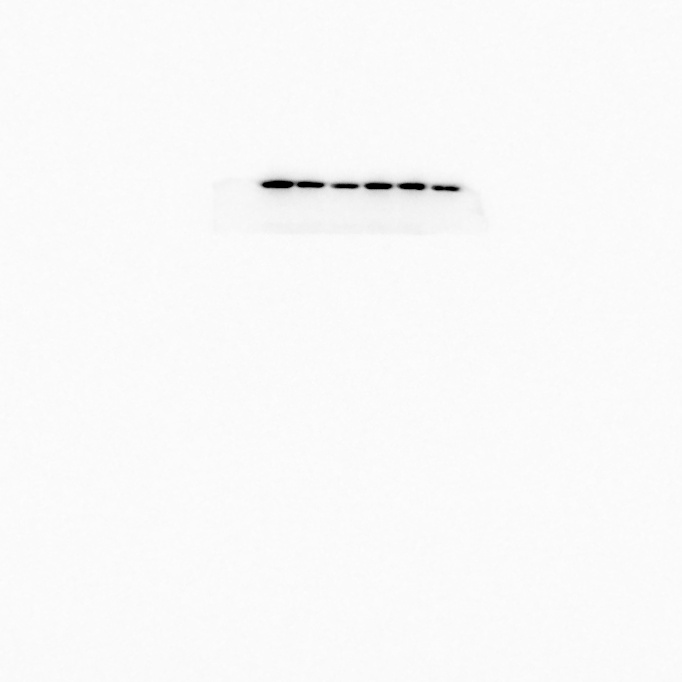

Supplement: Supplementary file 1 [file DataSheet_1.zip › original WB images/╬▓-actin-nig.tiff]

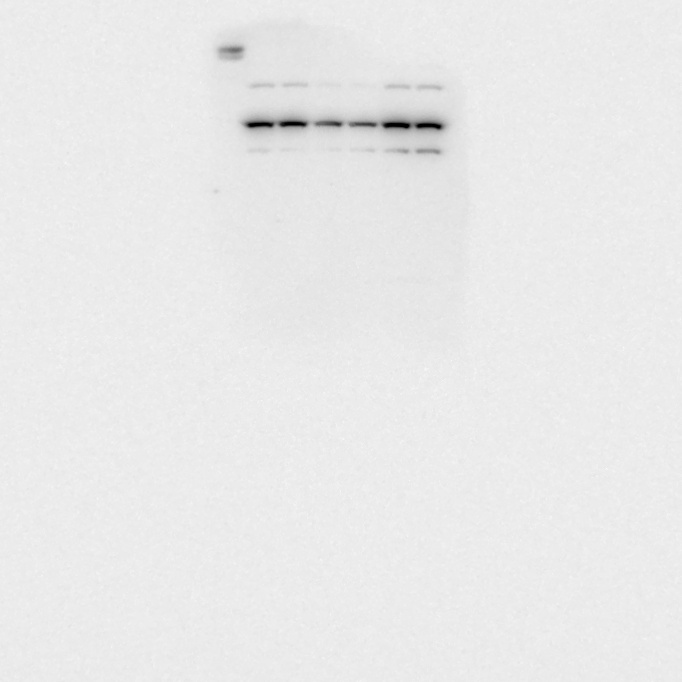

Supplement: Supplementary file 1 [file DataSheet_1.zip › original WB images/ATGL-ES.tiff]

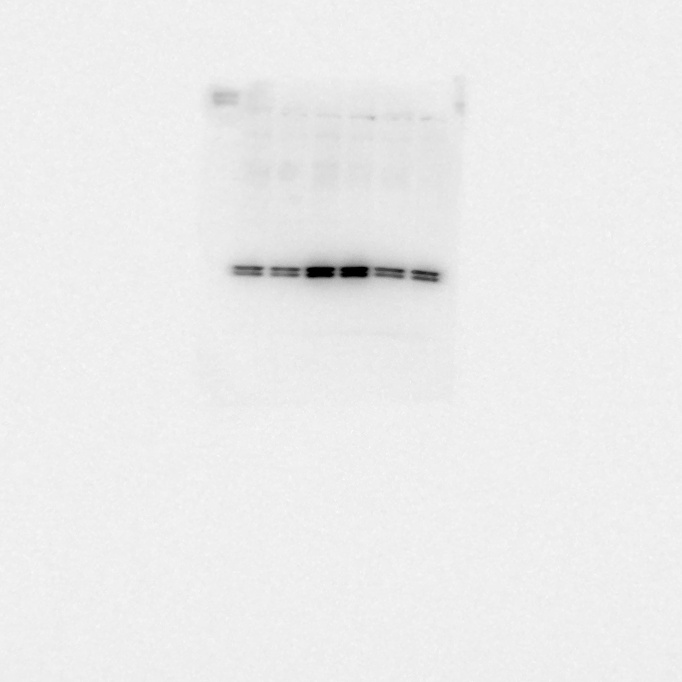

Supplement: Supplementary file 1 [file DataSheet_1.zip › original WB images/NLRP3-ES.tiff]

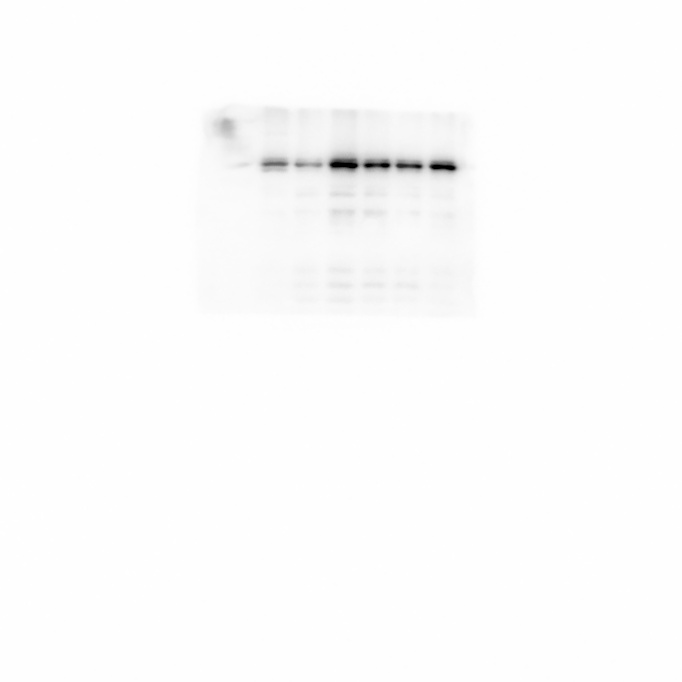

Supplement: Supplementary file 1 [file DataSheet_1.zip › original WB images/HSL-nig.tiff]

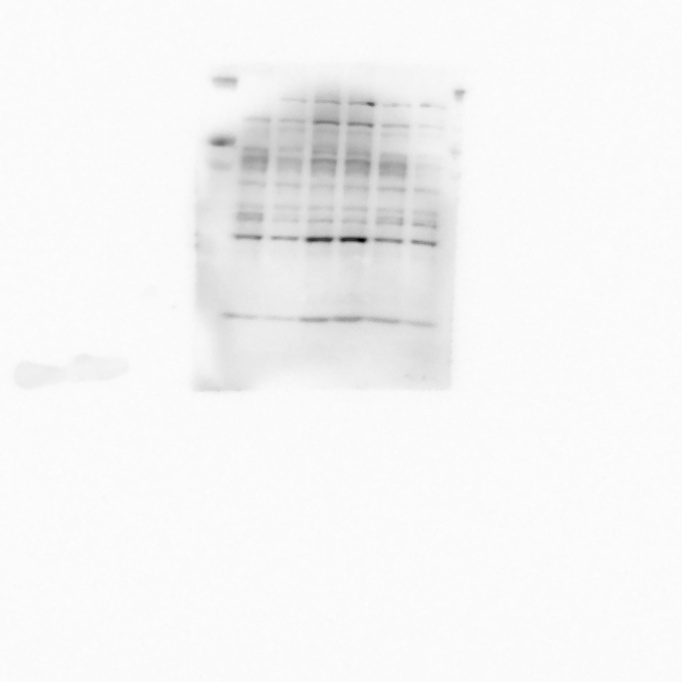

Supplement: Supplementary file 1 [file DataSheet_1.zip › original WB images/IL-1beta-ES.tiff]

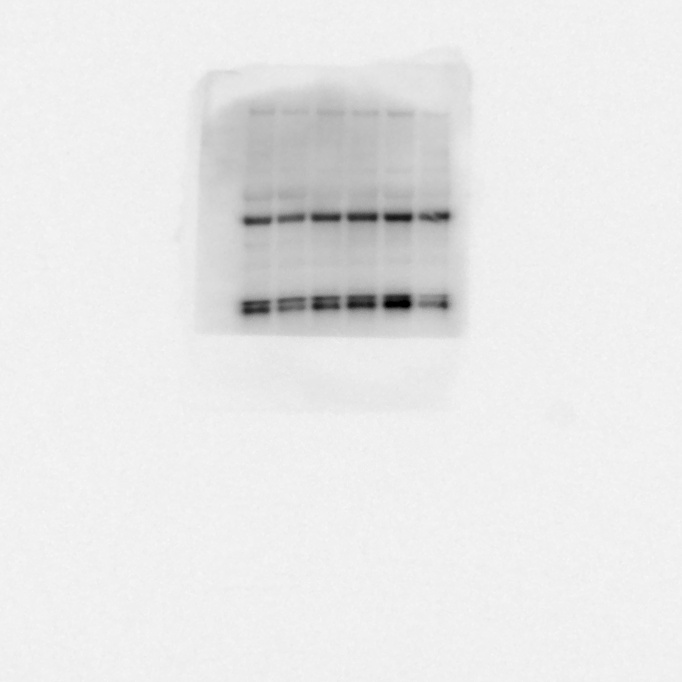

Supplement: Supplementary file 1 [file DataSheet_1.zip › original WB images/TH.tiff]

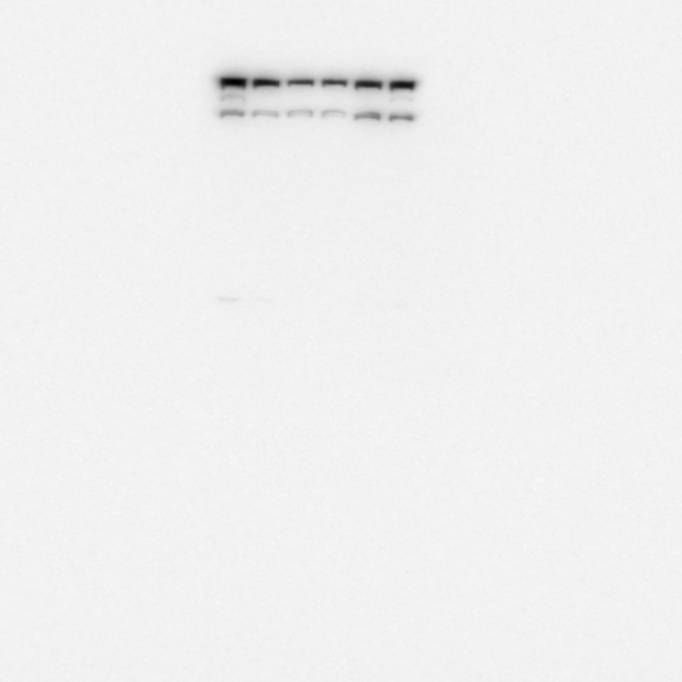

Supplement: Supplementary file 1 [file DataSheet_1.zip › original WB images/p-HSL-nig.tiff]

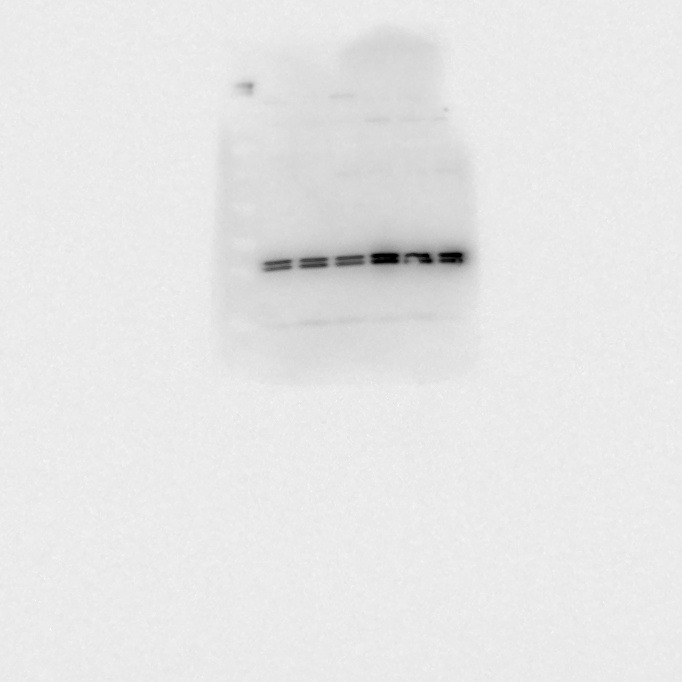

Supplement: Supplementary file 1 [file DataSheet_1.zip › original WB images/NLRP3-nig.tiff]

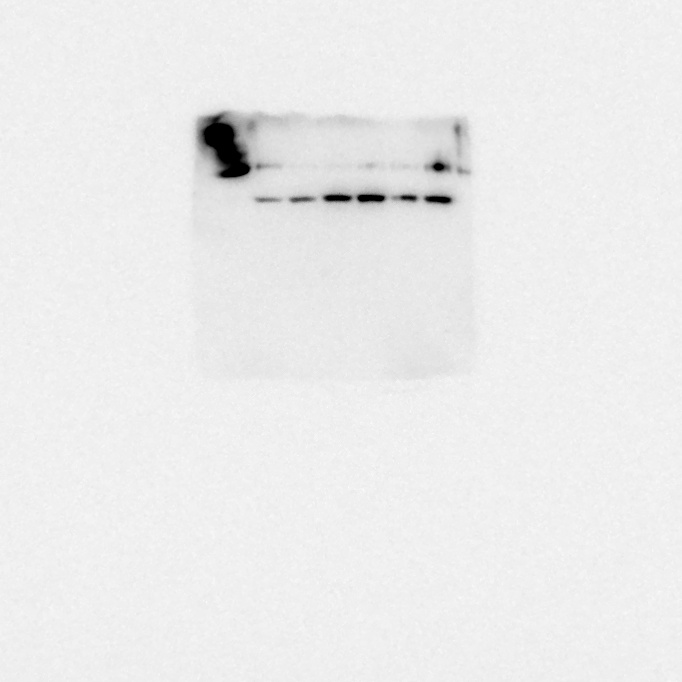

Supplement: Supplementary file 1 [file DataSheet_1.zip › original WB images/MAO-Nig.tiff]

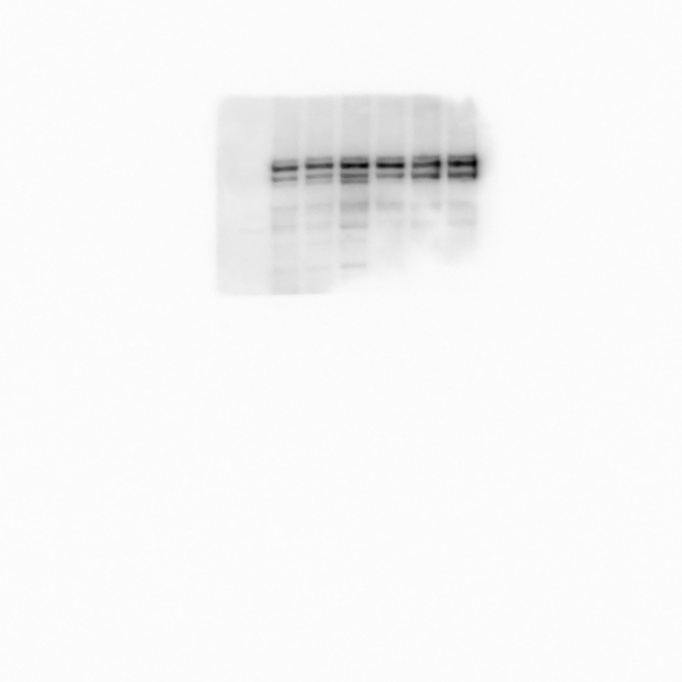

Supplement: Supplementary file 1 [file DataSheet_1.zip › original WB images/HSL-ES.tiff]

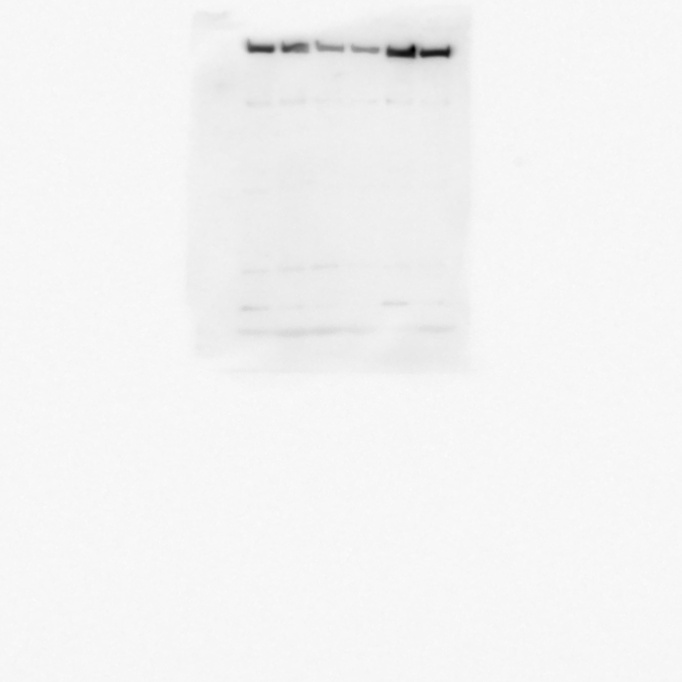

Supplement: Supplementary file 1 [file DataSheet_1.zip › original WB images/p-HSL-ES.tiff]

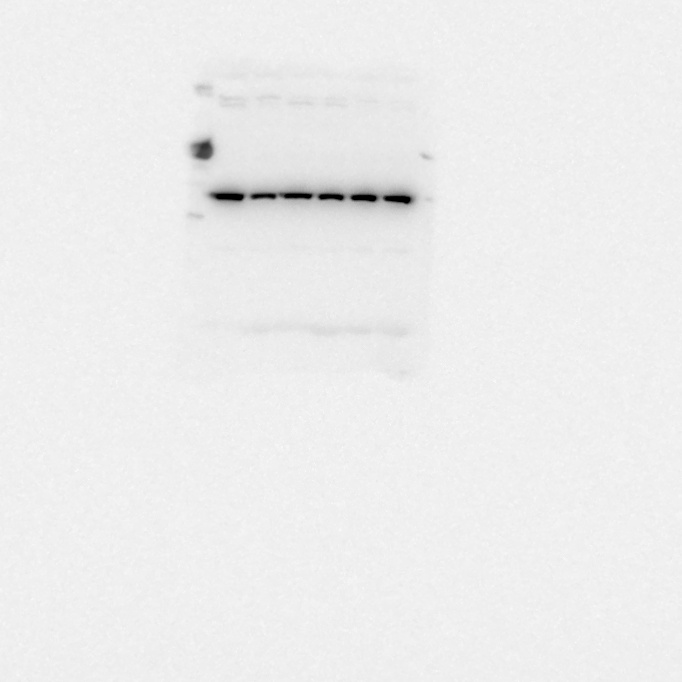

Supplement: Supplementary file 1 [file DataSheet_1.zip › original WB images/╬▓-actin-Es.tiff]

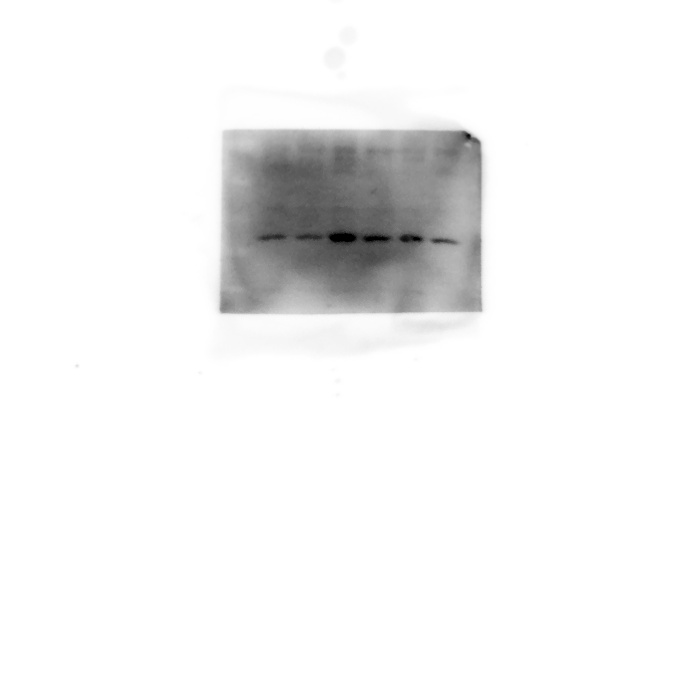

Supplement: Supplementary file 1 [file DataSheet_1.zip › original WB images/caspase1-nig.tiff]

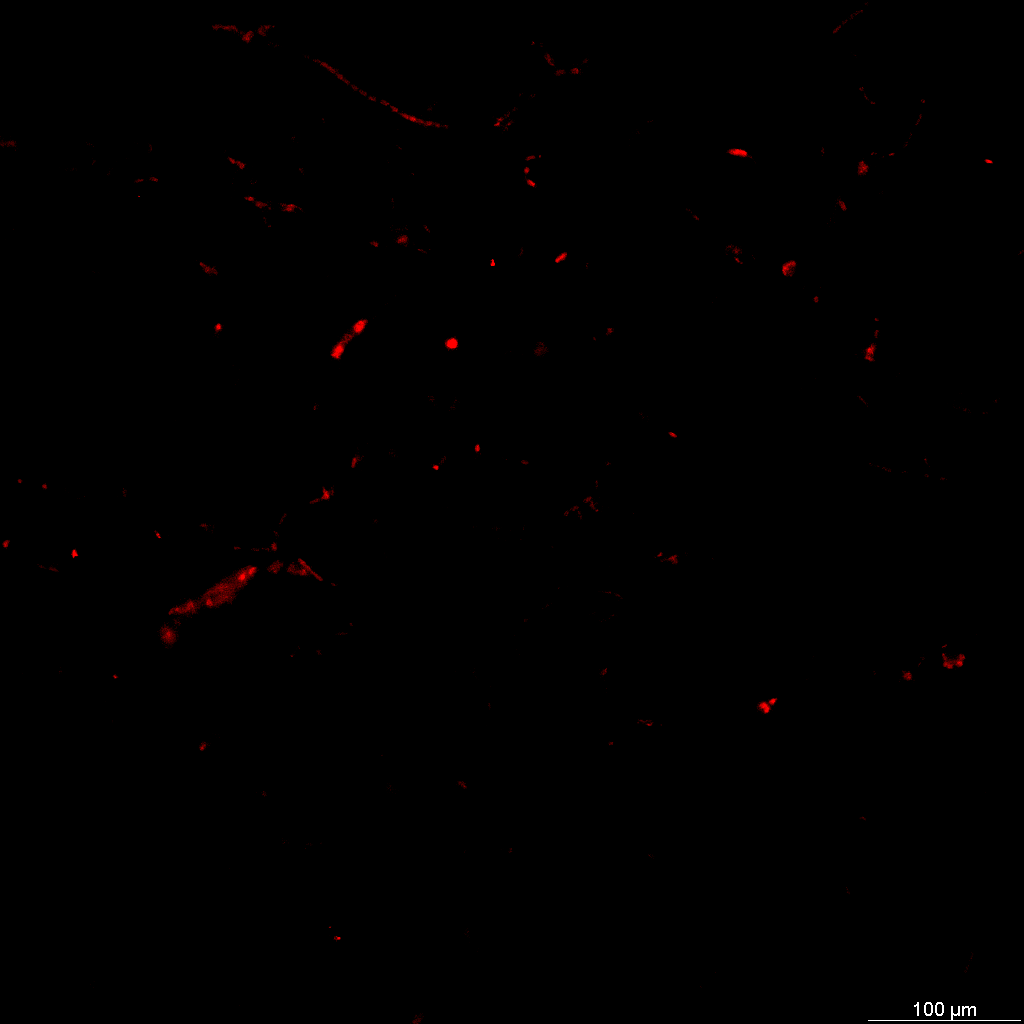

Supplement: Supplementary file 2 [file DataSheet_2.zip › Project002_Series023_Lng_LVCC_Processed001_ch02.tif]

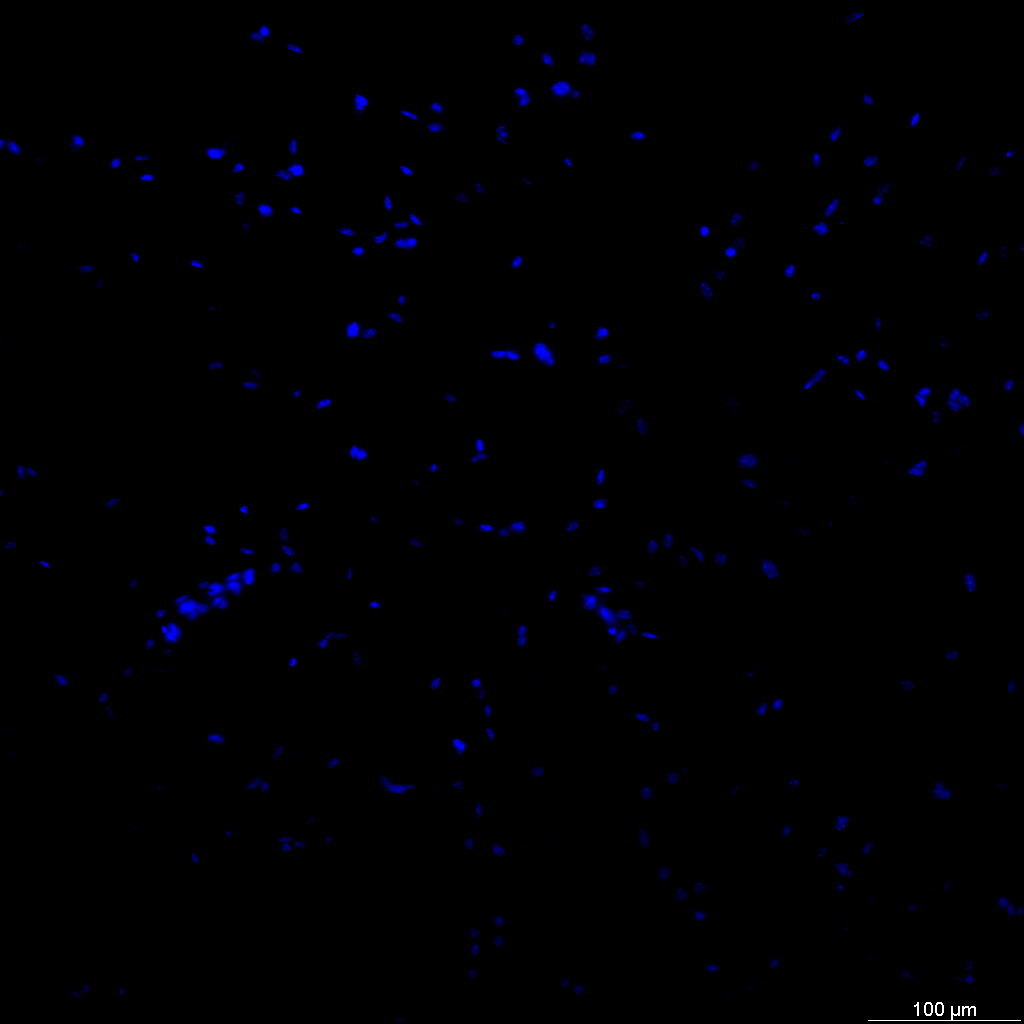

Supplement: Supplementary file 2 [file DataSheet_2.zip › Project002_Series023_Lng_LVCC_Processed001_ch00.tif]

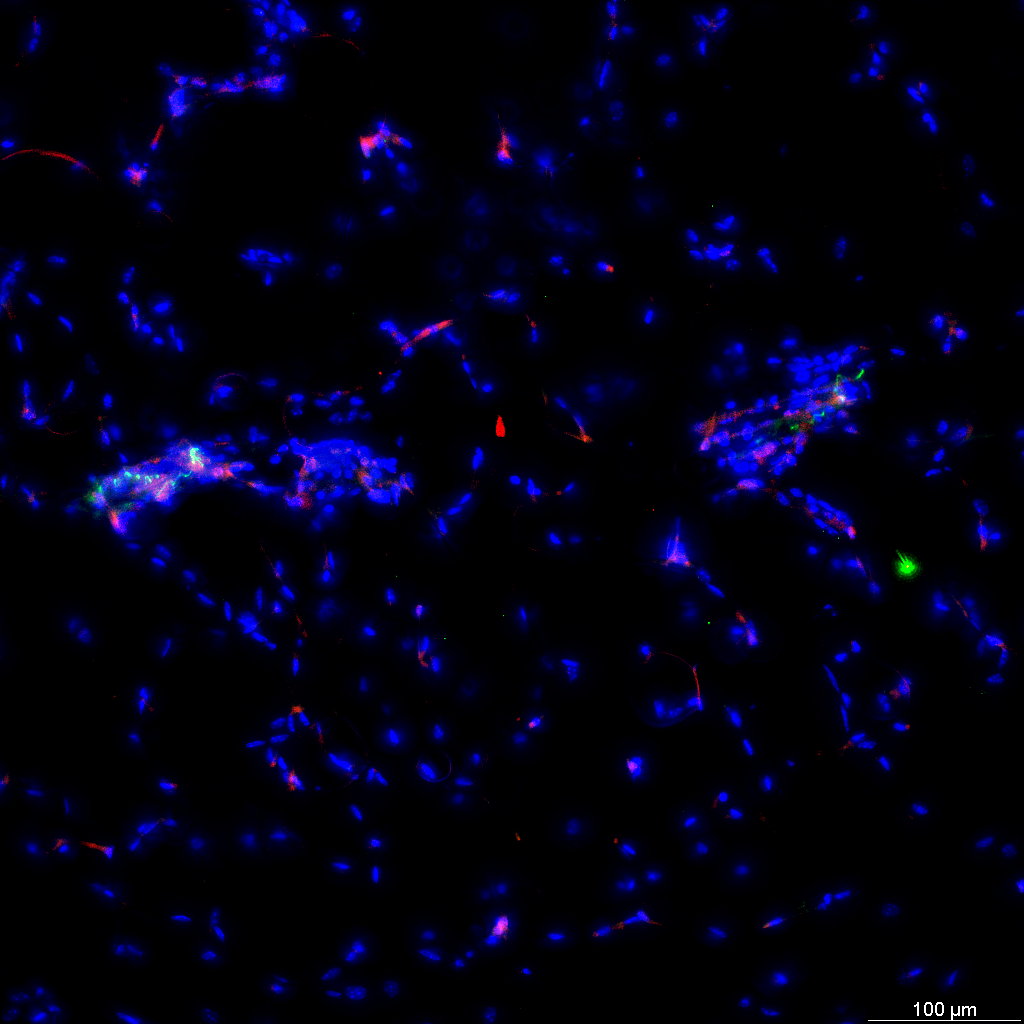

Supplement: Supplementary file 2 [file DataSheet_2.zip › Project002_Series080_Lng_LVCC_Processed001.tif]

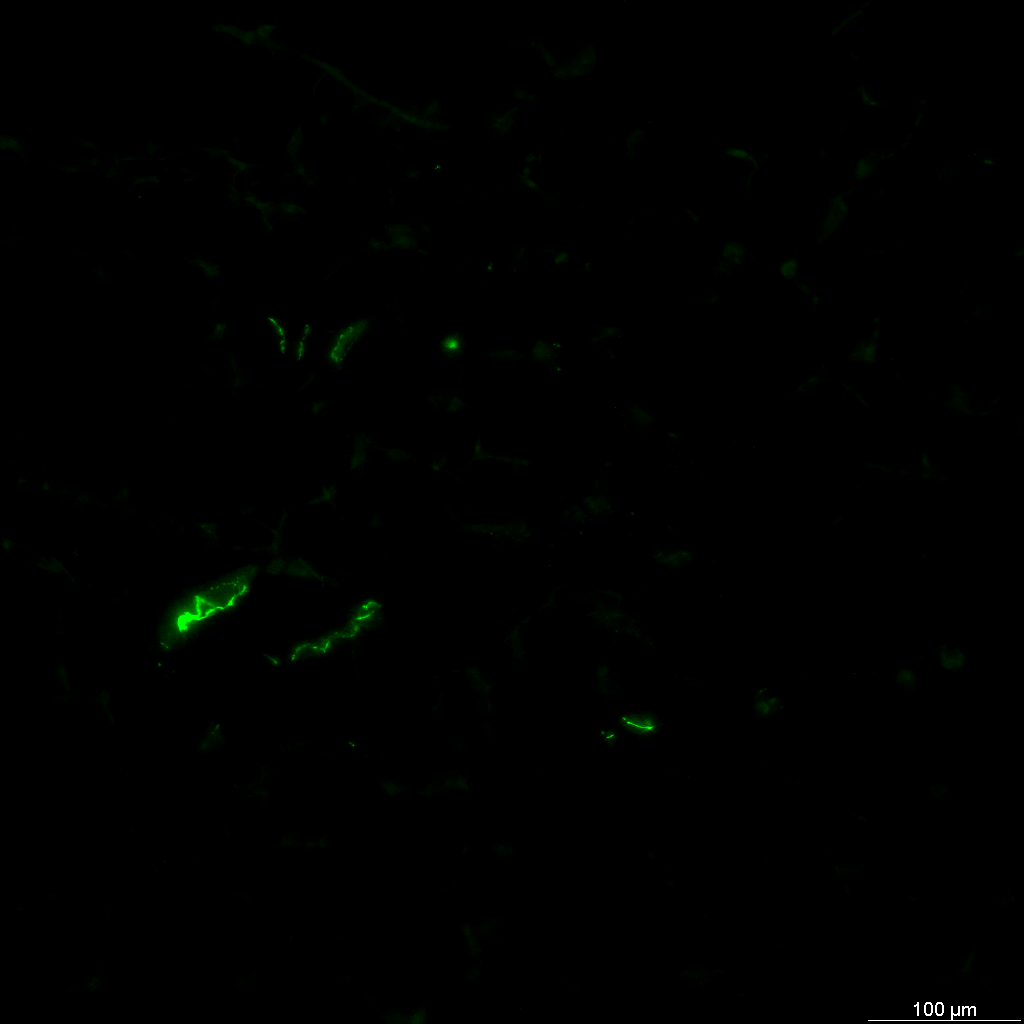

Supplement: Supplementary file 2 [file DataSheet_2.zip › Project002_Series023_Lng_LVCC_Processed001_ch01.tif]

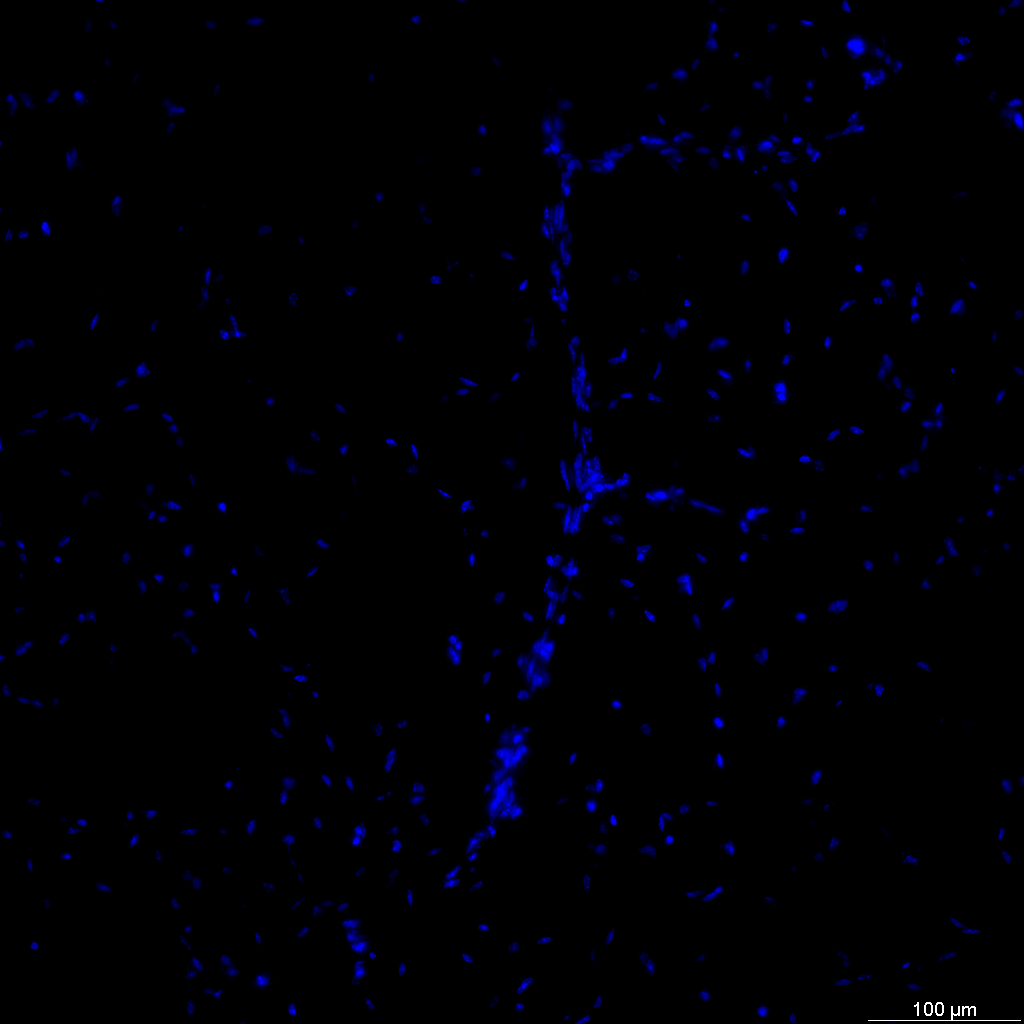

Supplement: Supplementary file 2 [file DataSheet_2.zip › Project002_Series075_Lng_LVCC_Processed001dapi_ch00.tif]

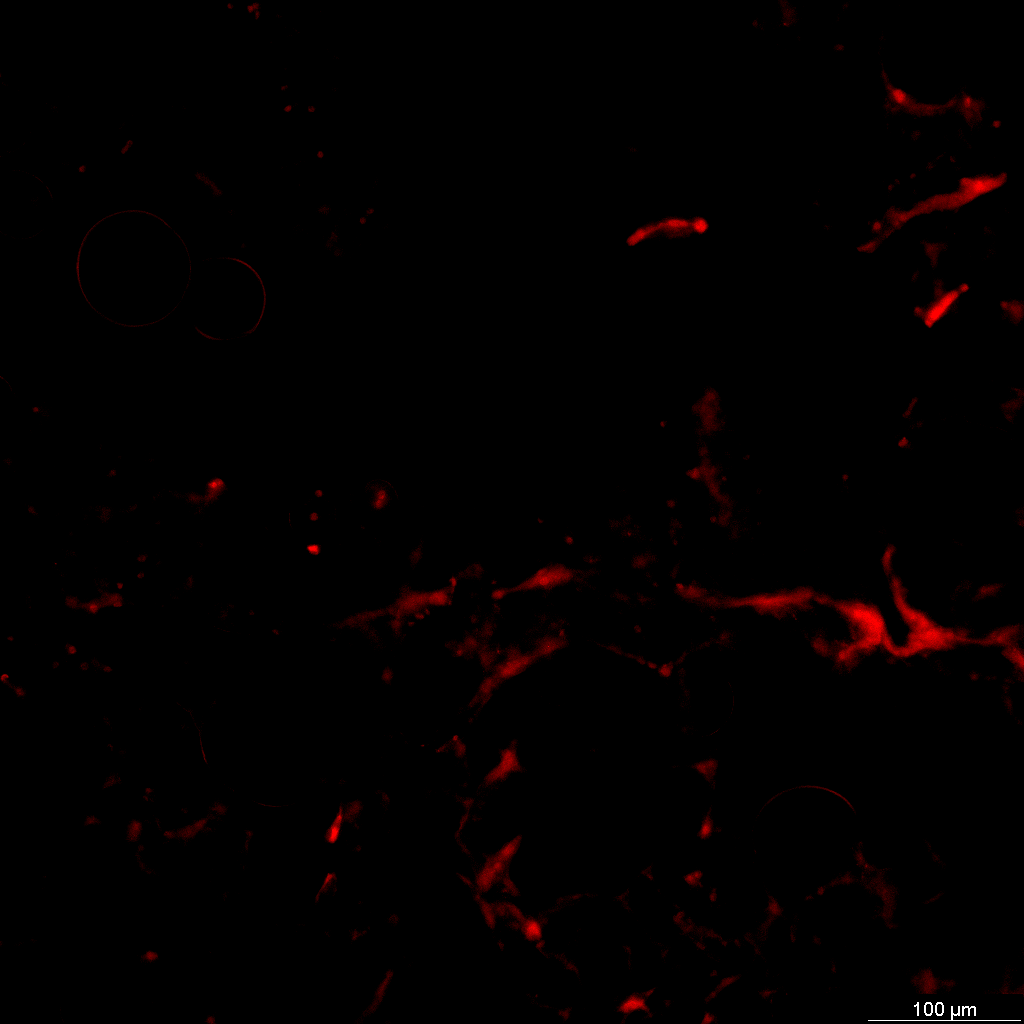

Supplement: Supplementary file 2 [file DataSheet_2.zip › Project0020210604_Series024_Lng_LVCC_Processed001_ch02.tif]

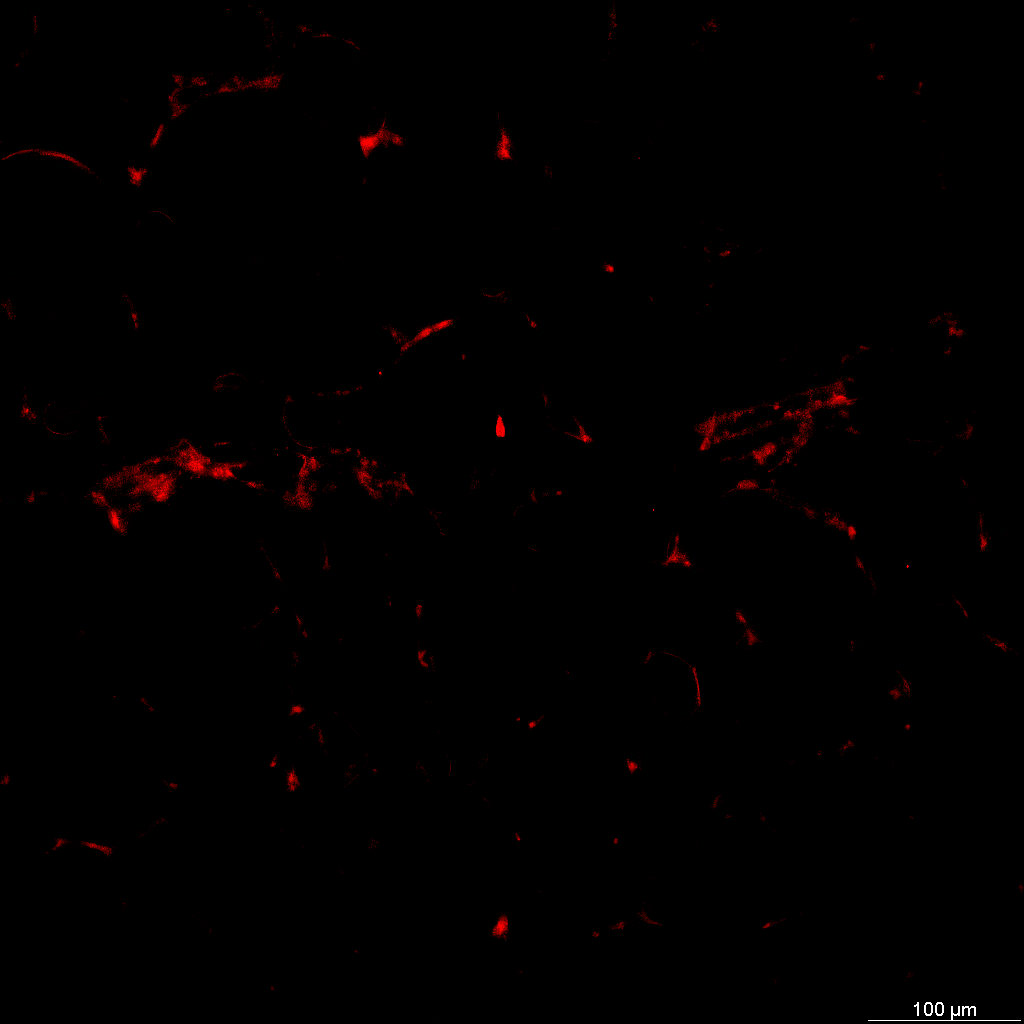

Supplement: Supplementary file 2 [file DataSheet_2.zip › Project002_Series080_Lng_LVCC_Processed001_ch02.tif]

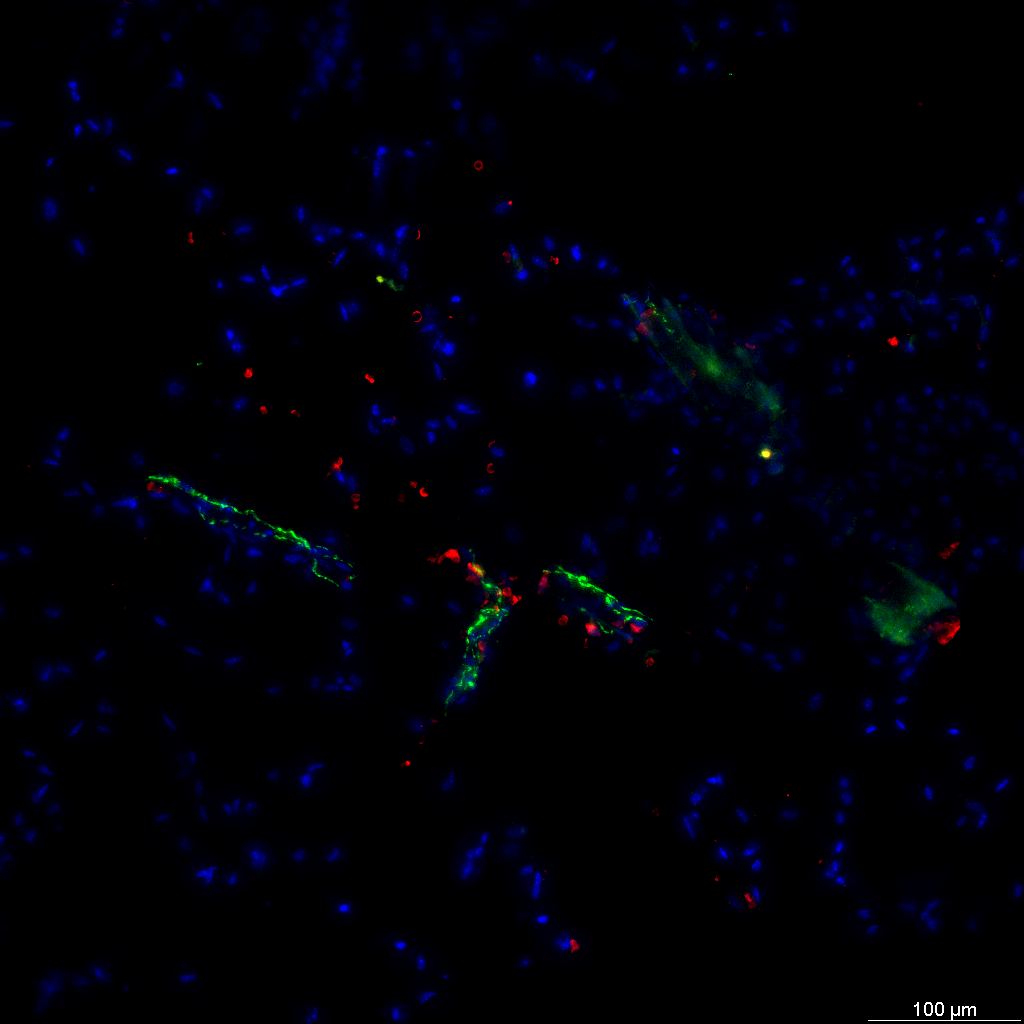

Supplement: Supplementary file 2 [file DataSheet_2.zip › Project004_Series037_Lng_LVCC_Processed001.tif]

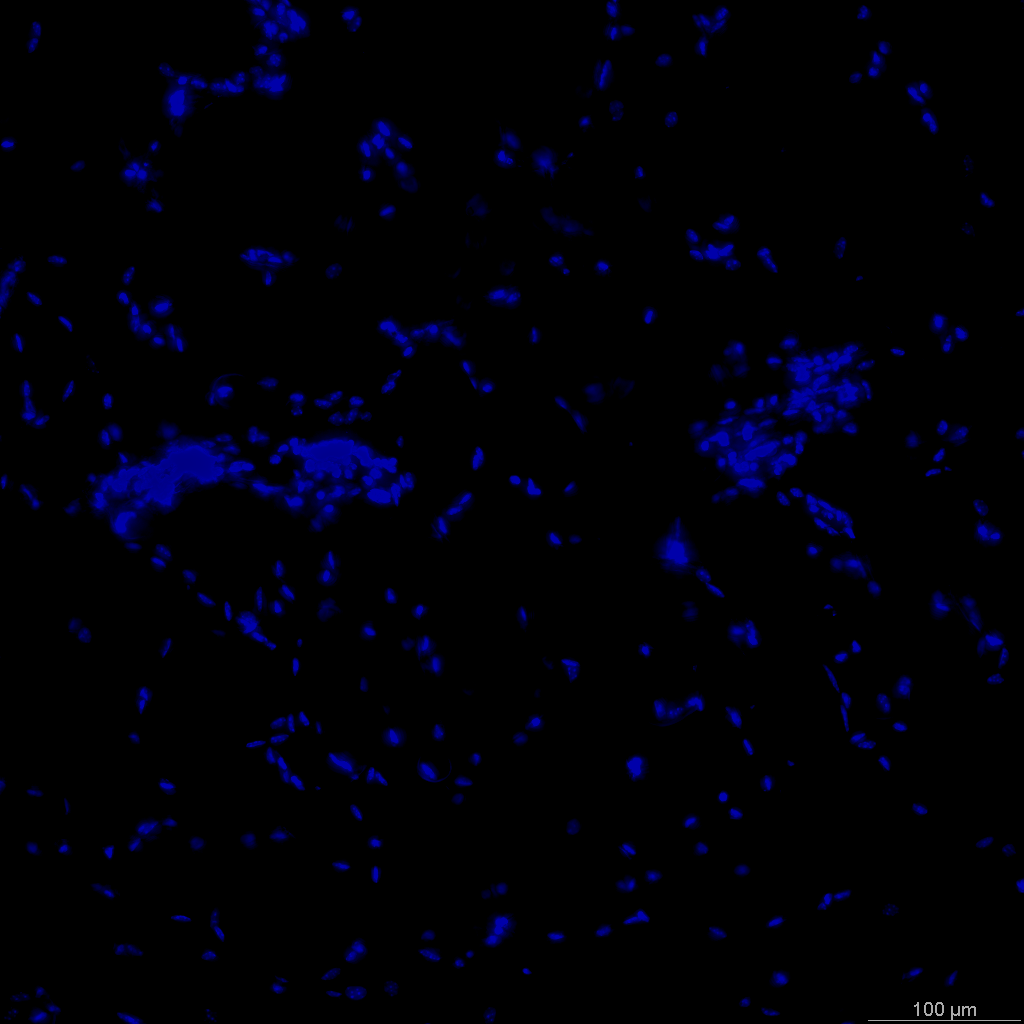

Supplement: Supplementary file 2 [file DataSheet_2.zip › Project002_Series080_Lng_LVCC_Processed001_ch00.tif]

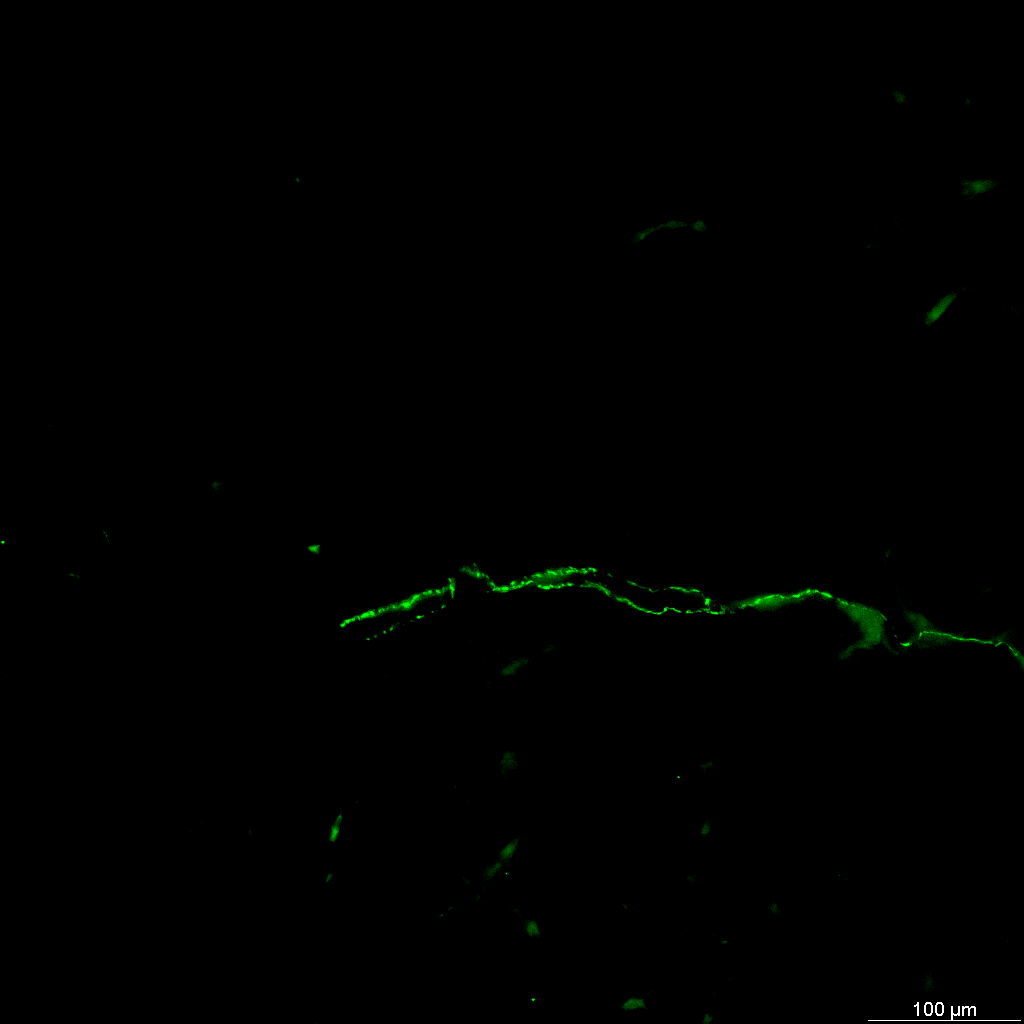

Supplement: Supplementary file 2 [file DataSheet_2.zip › Project0020210604_Series024_Lng_LVCC_Processed001_ch01.tif]

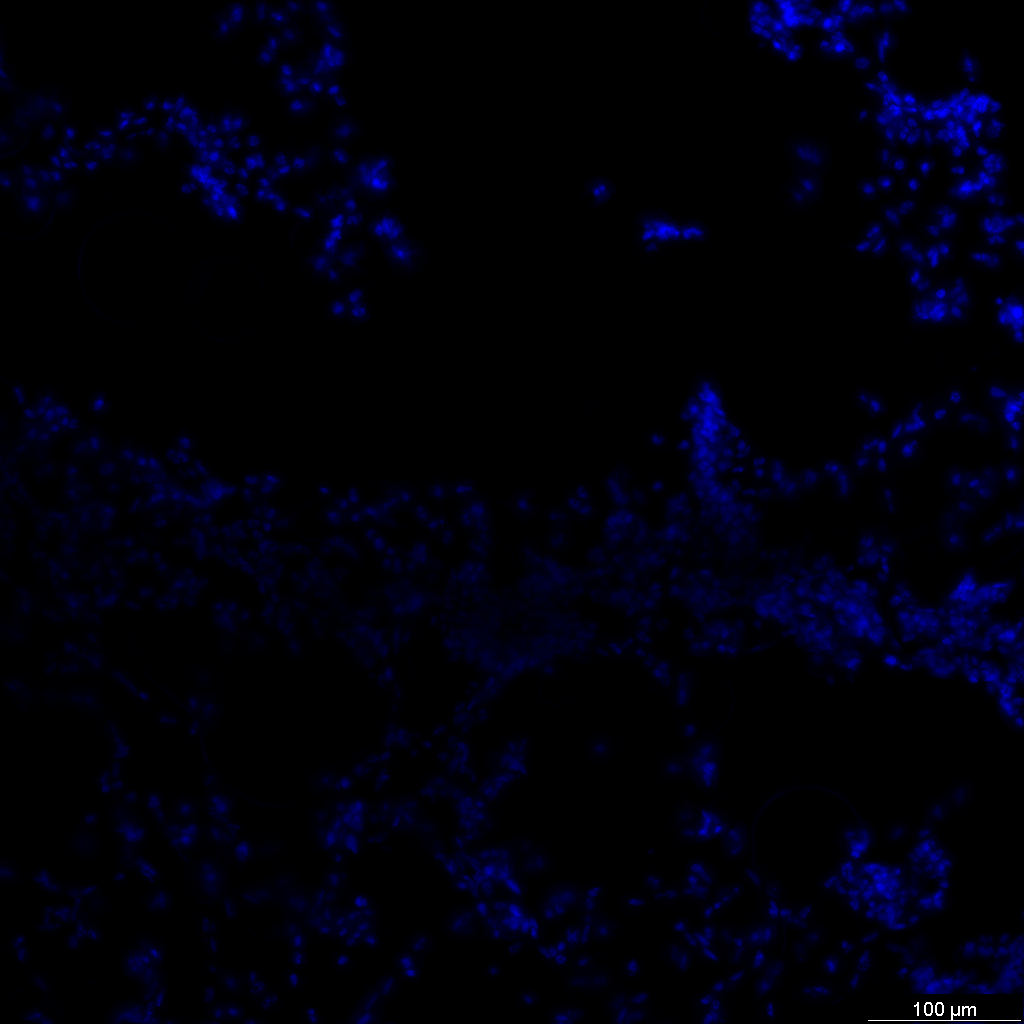

Supplement: Supplementary file 2 [file DataSheet_2.zip › Project0020210604_Series024_Lng_LVCC_Processed001_ch00.tif]

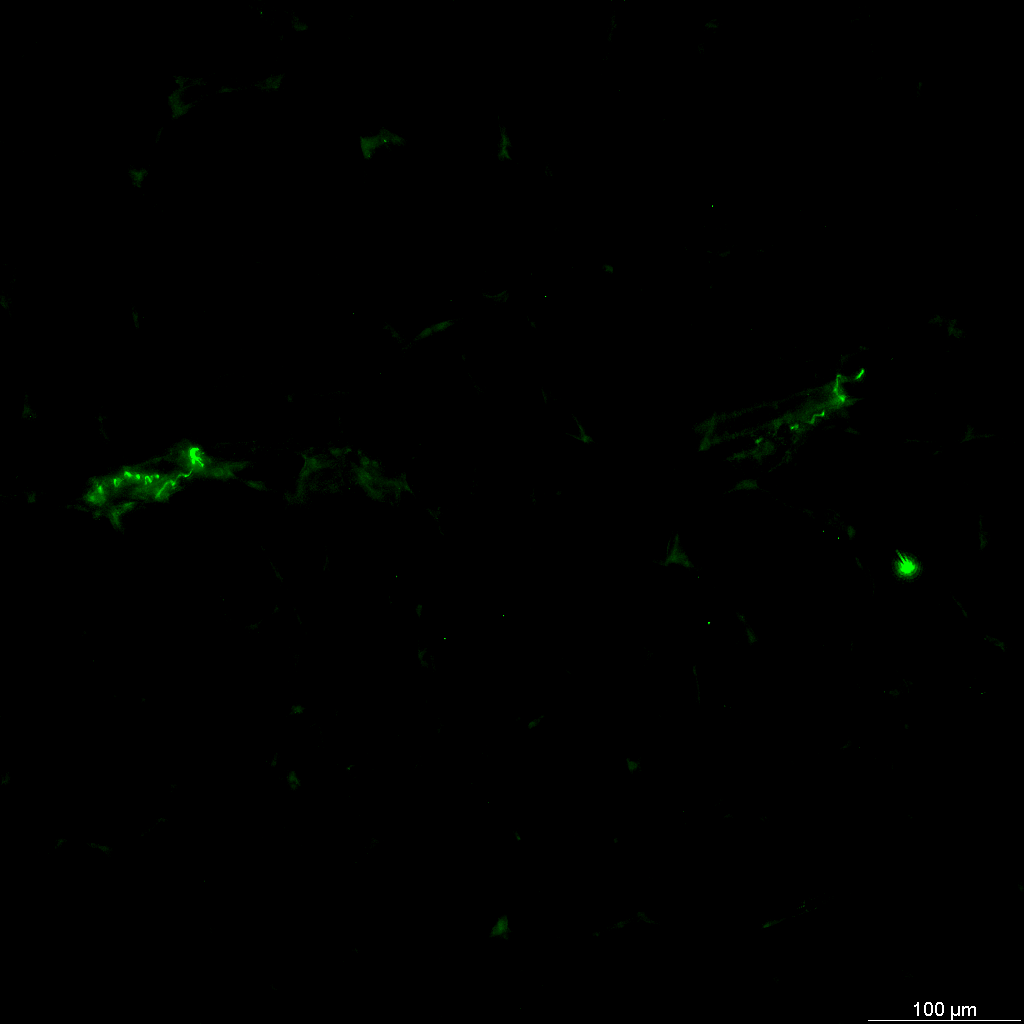

Supplement: Supplementary file 2 [file DataSheet_2.zip › Project002_Series080_Lng_LVCC_Processed001_ch01.tif]

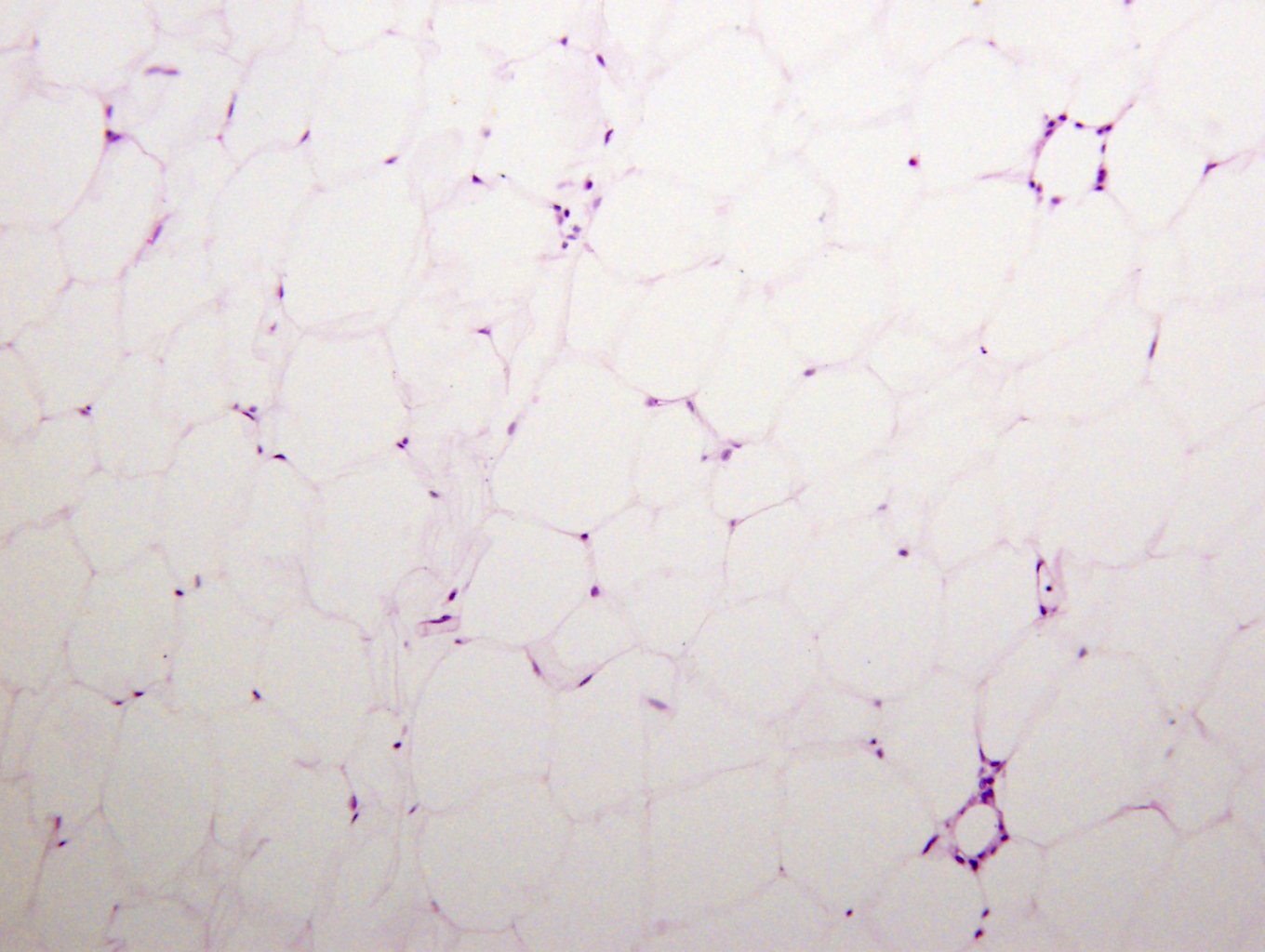

Supplement: Supplementary file 2 [file DataSheet_2.zip › HFD.jpg]

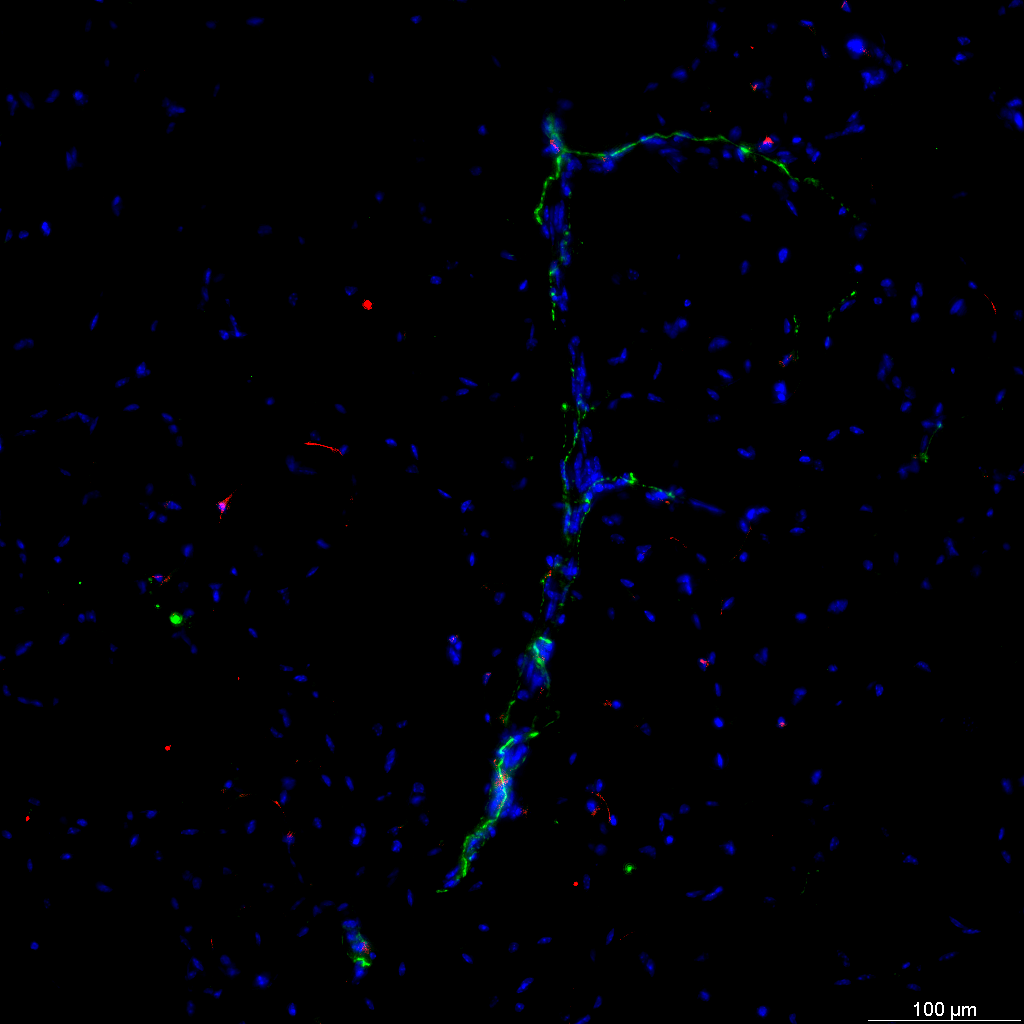

Supplement: Supplementary file 2 [file DataSheet_2.zip › Project002_Series075_Lng_LVCC_Processed001merge.tif]

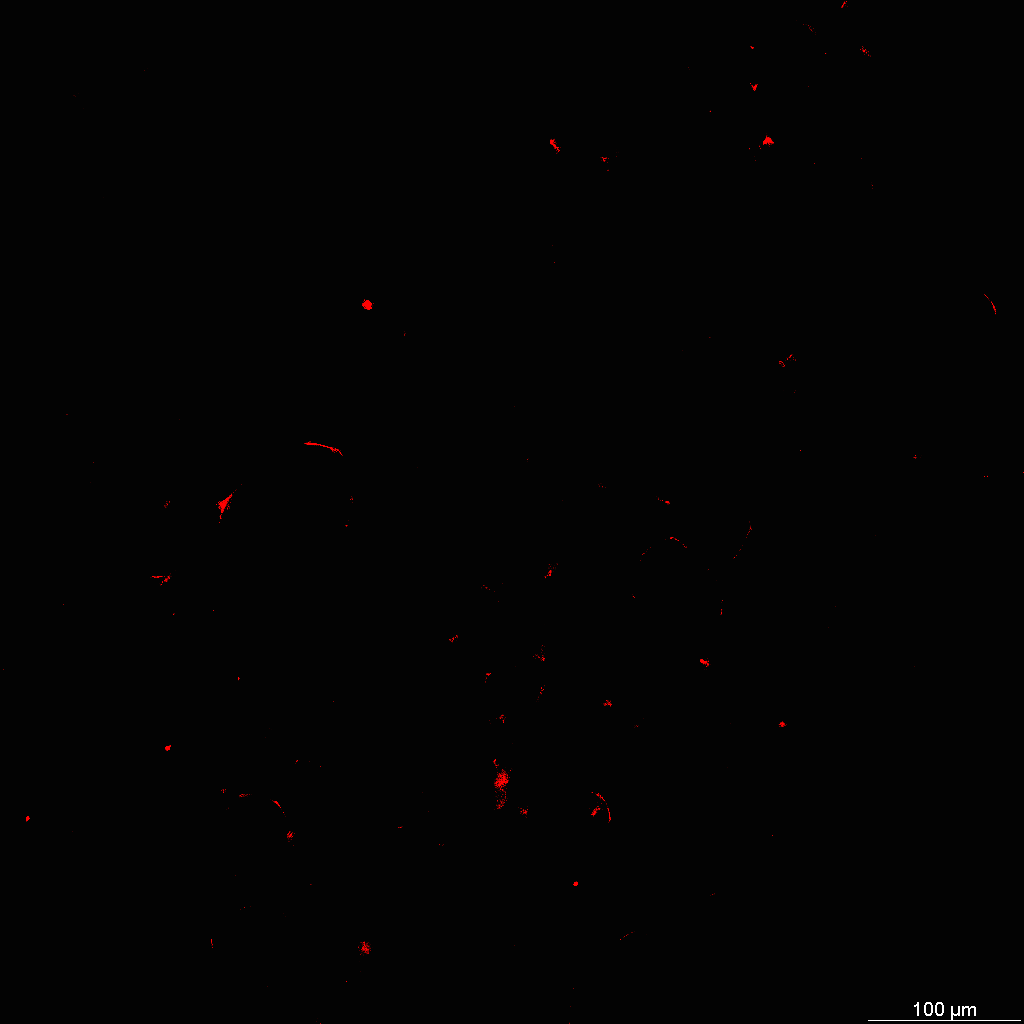

Supplement: Supplementary file 2 [file DataSheet_2.zip › Project002_Series075_Lng_LVCC_Processed001f480_ch02.tif]

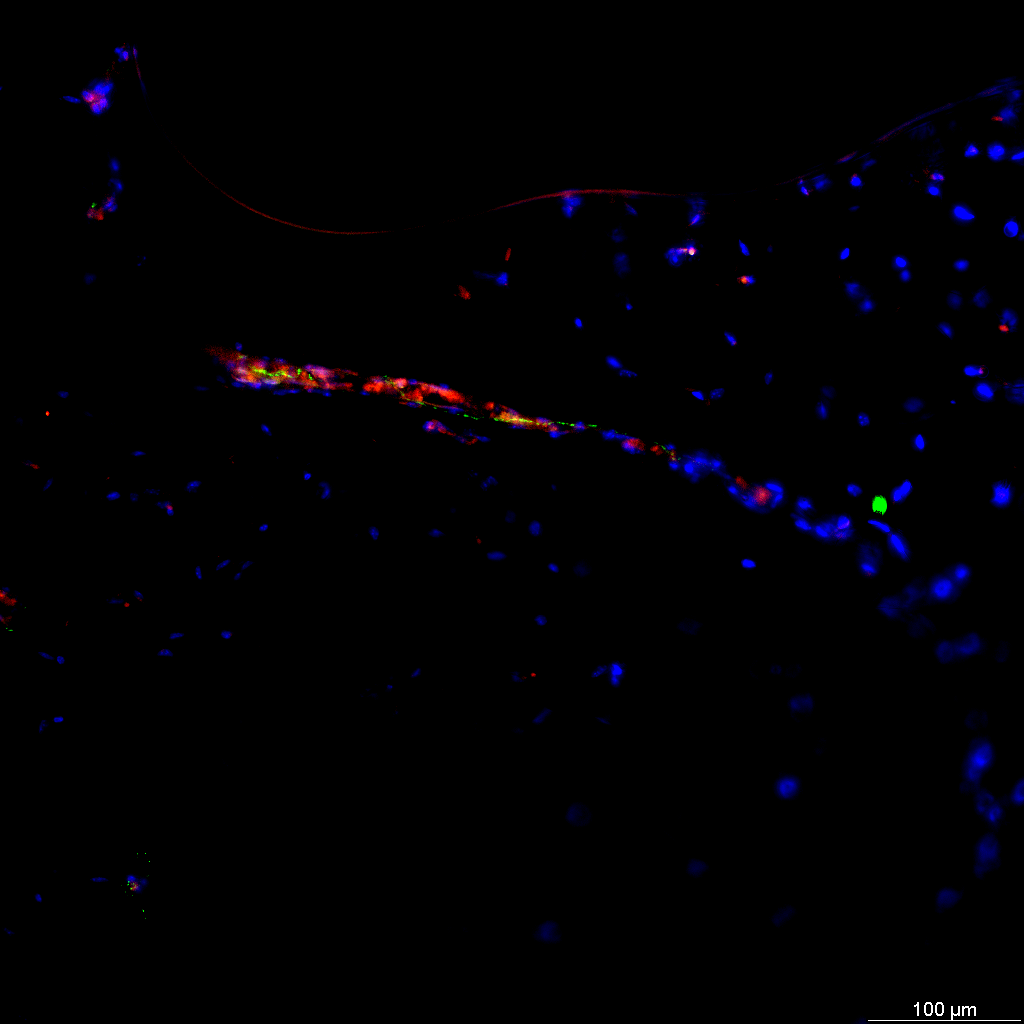

Supplement: Supplementary file 2 [file DataSheet_2.zip › Project004_Series025_Lng_LVCC_Processed001.tif]

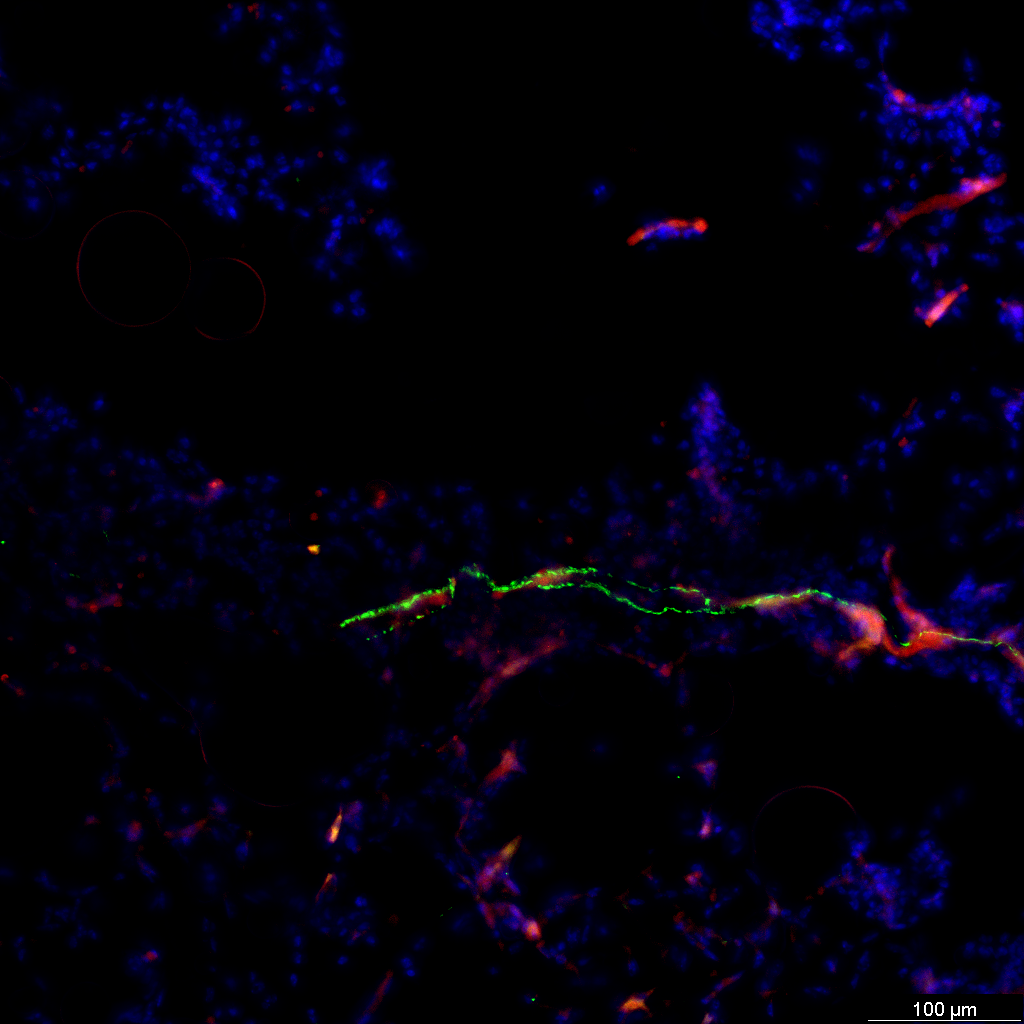

Supplement: Supplementary file 2 [file DataSheet_2.zip › Project0020210604_Series024_Lng_LVCC_Processed001.tif]

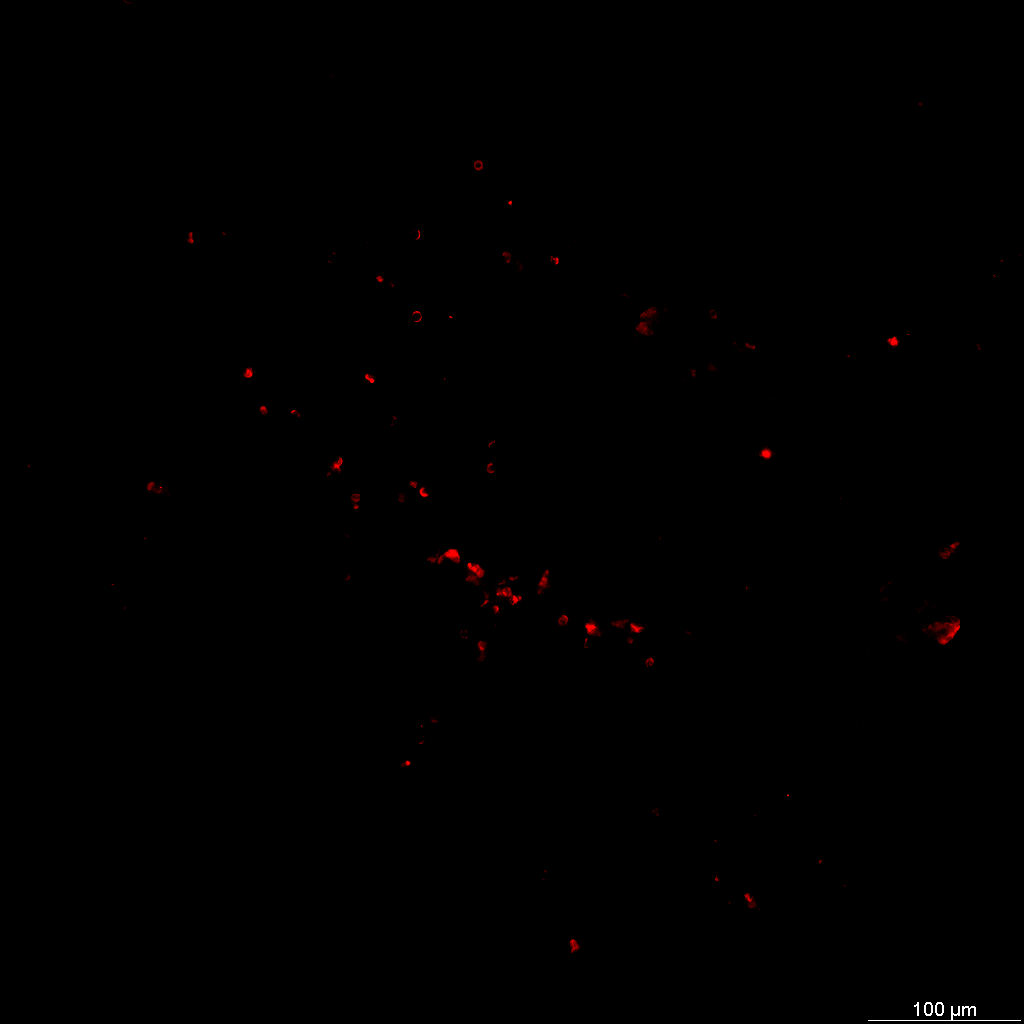

Supplement: Supplementary file 2 [file DataSheet_2.zip › Project004_Series037_Lng_LVCC_Processed001_ch02.tif]

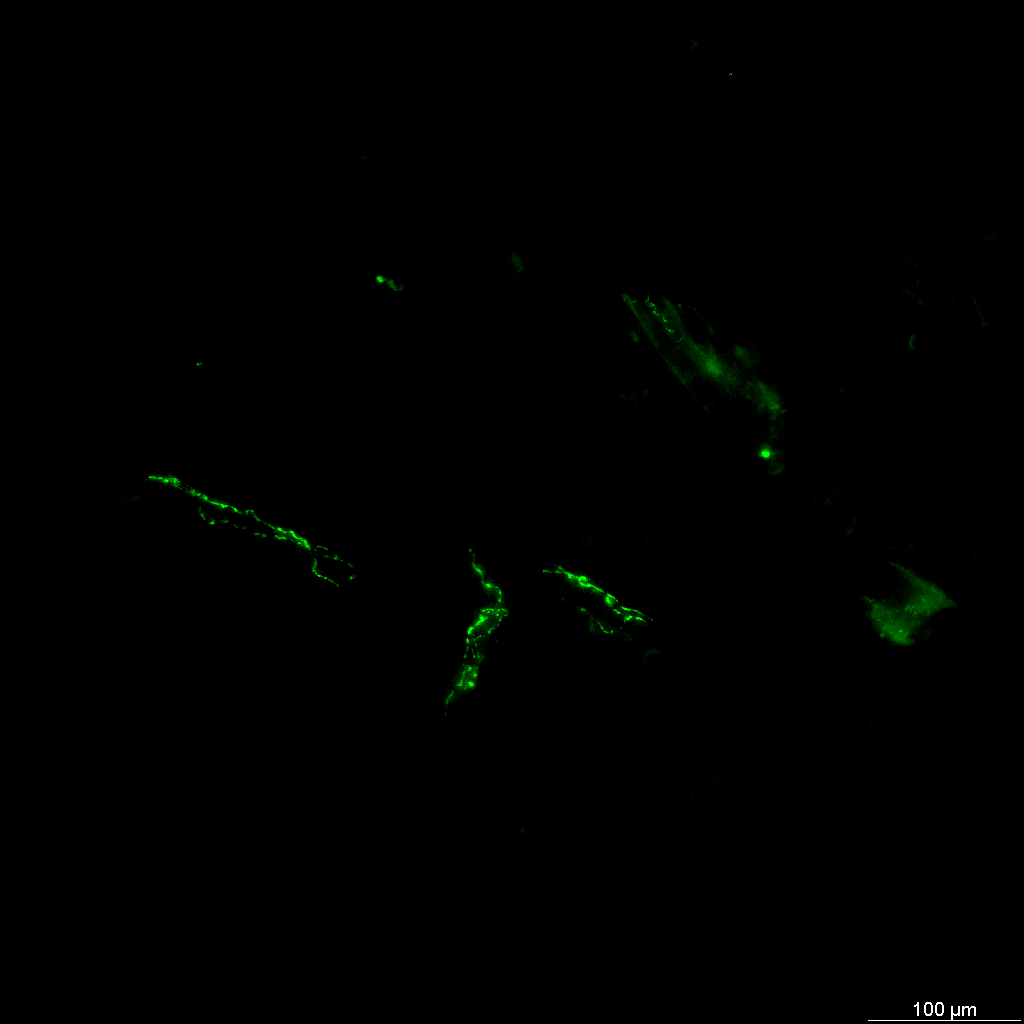

Supplement: Supplementary file 2 [file DataSheet_2.zip › Project004_Series037_Lng_LVCC_Processed001_ch01.tif]

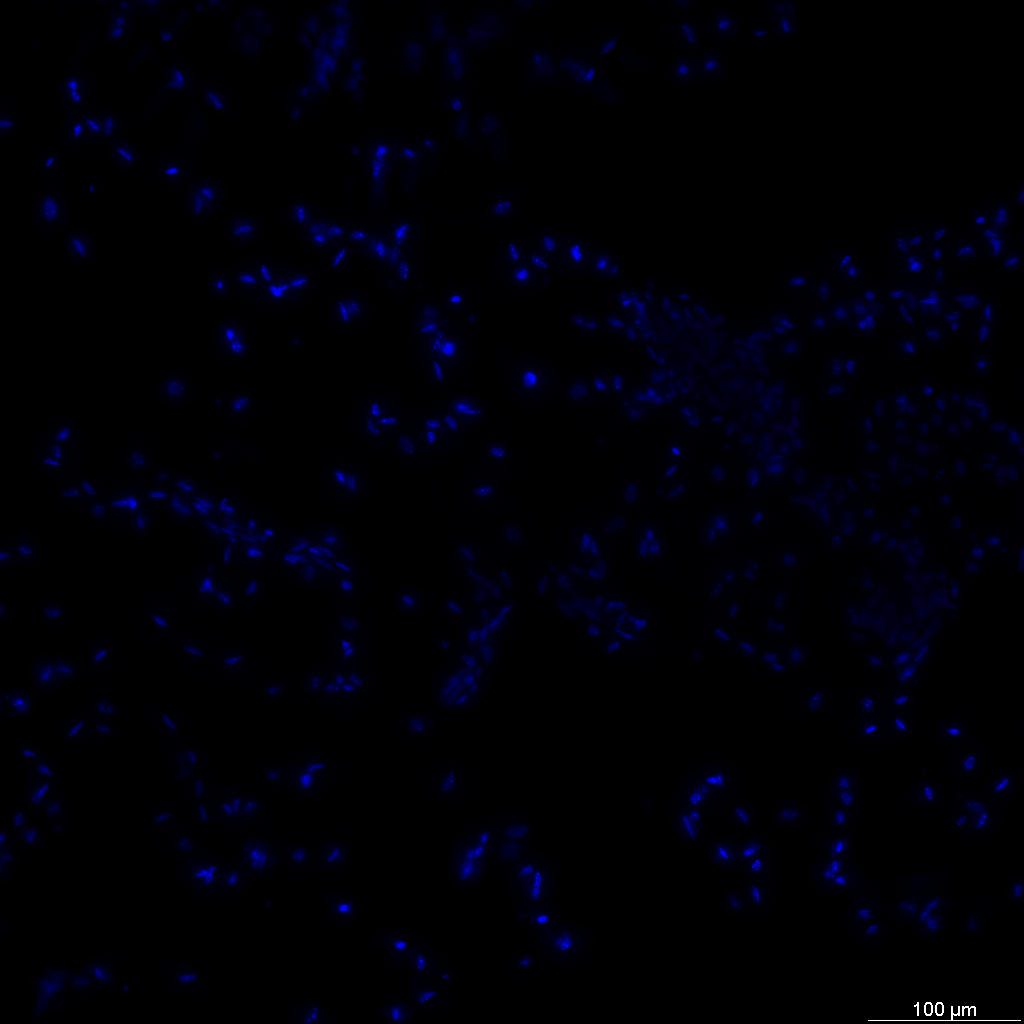

Supplement: Supplementary file 2 [file DataSheet_2.zip › Project004_Series037_Lng_LVCC_Processed001_ch00.tif]

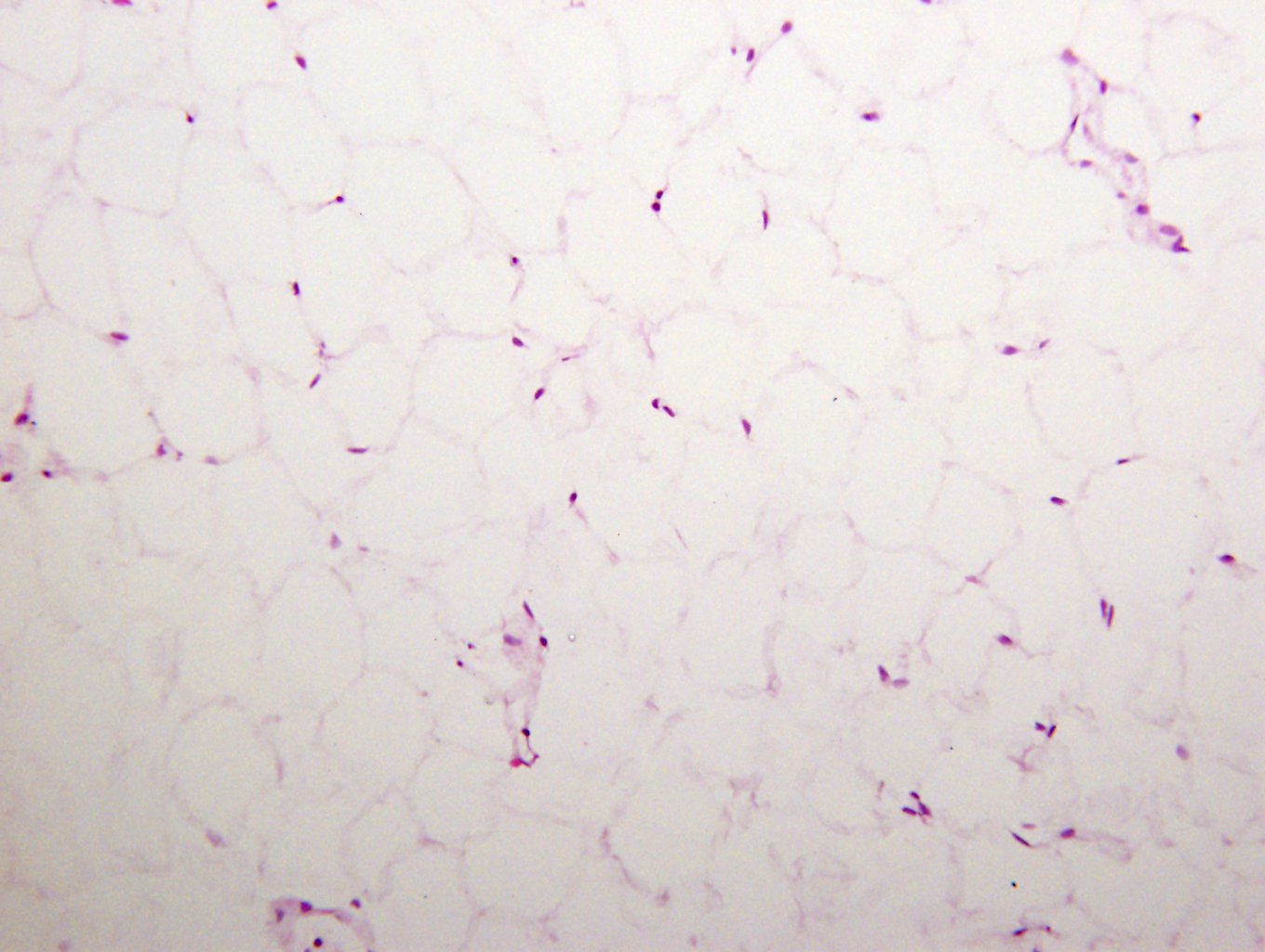

Supplement: Supplementary file 2 [file DataSheet_2.zip › HFD+ES.jpg]

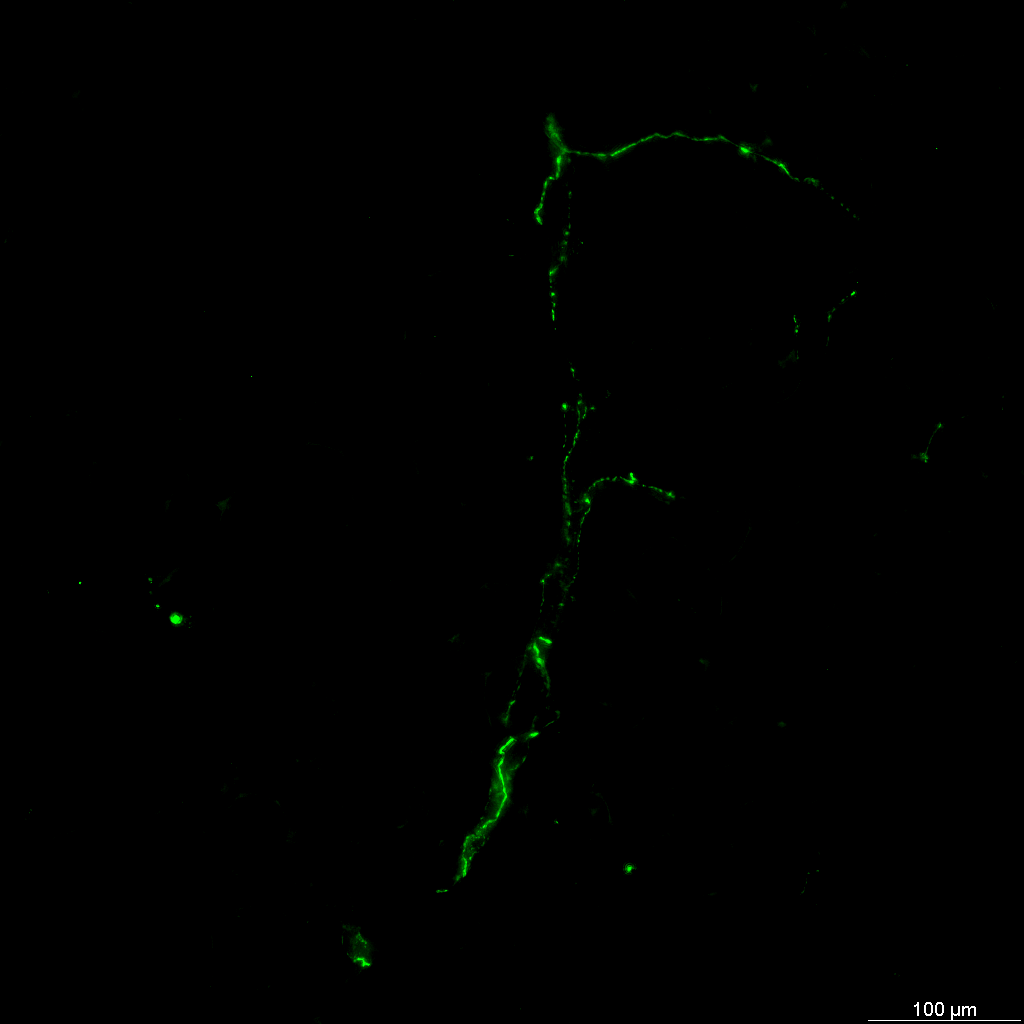

Supplement: Supplementary file 2 [file DataSheet_2.zip › Project002_Series075_Lng_LVCC_Processed001th_ch01.tif]

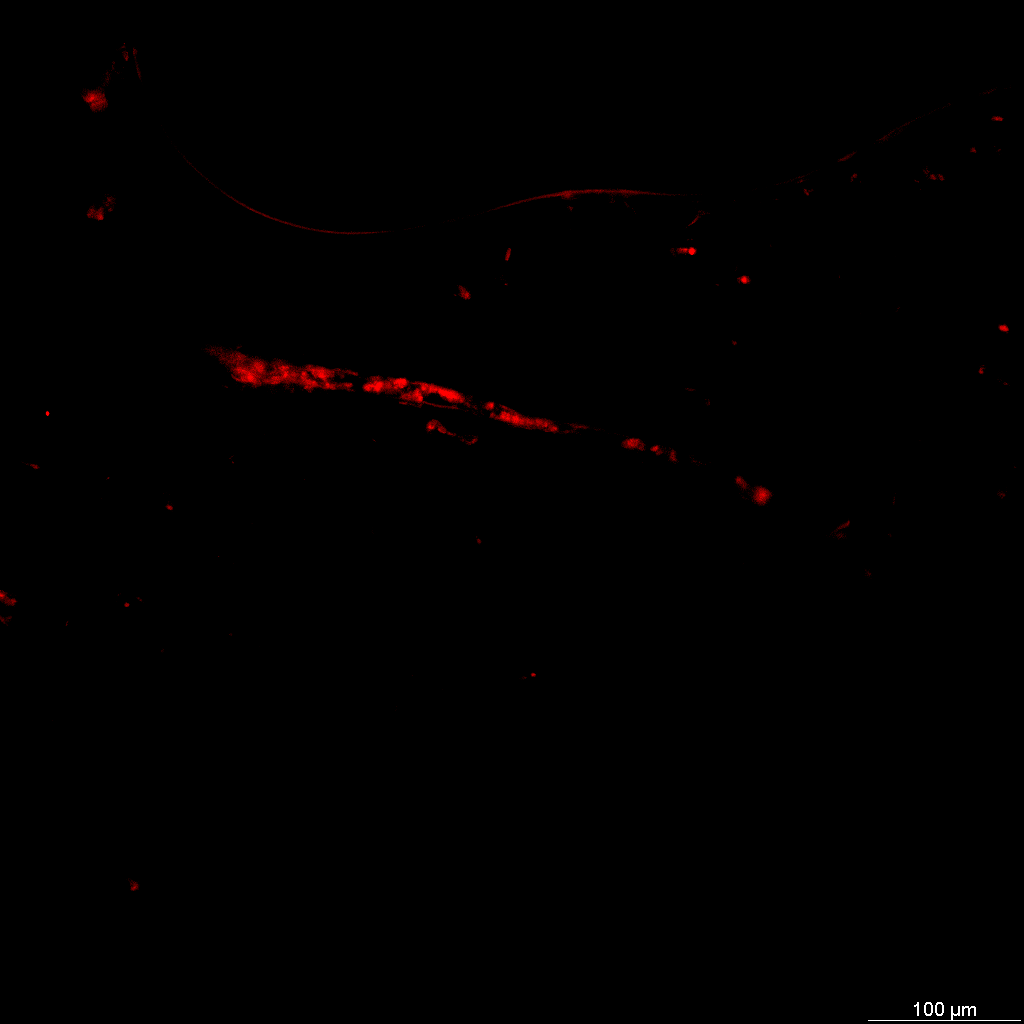

Supplement: Supplementary file 2 [file DataSheet_2.zip › Project004_Series025_Lng_LVCC_Processed001_ch02.tif]

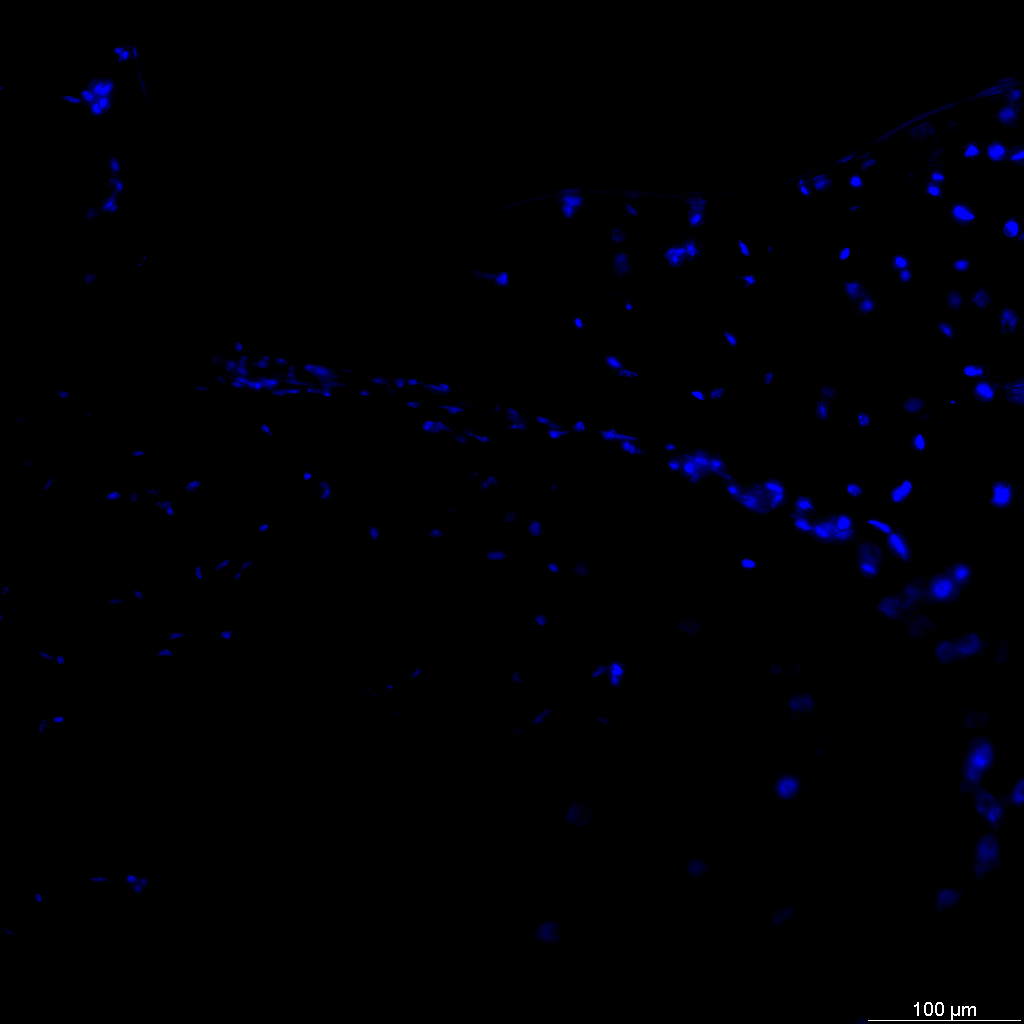

Supplement: Supplementary file 2 [file DataSheet_2.zip › Project004_Series025_Lng_LVCC_Processed001_ch00.tif]

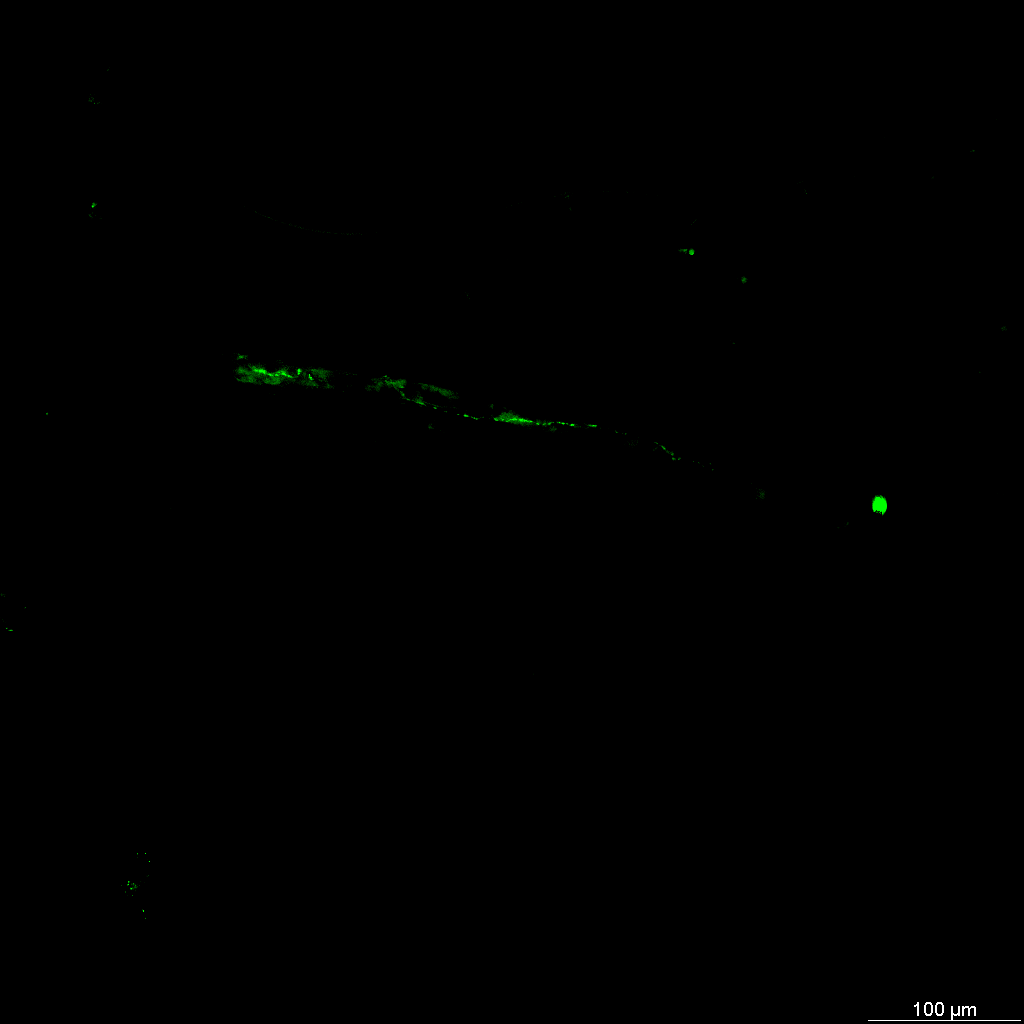

Supplement: Supplementary file 2 [file DataSheet_2.zip › Project004_Series025_Lng_LVCC_Processed001_ch01.tif]

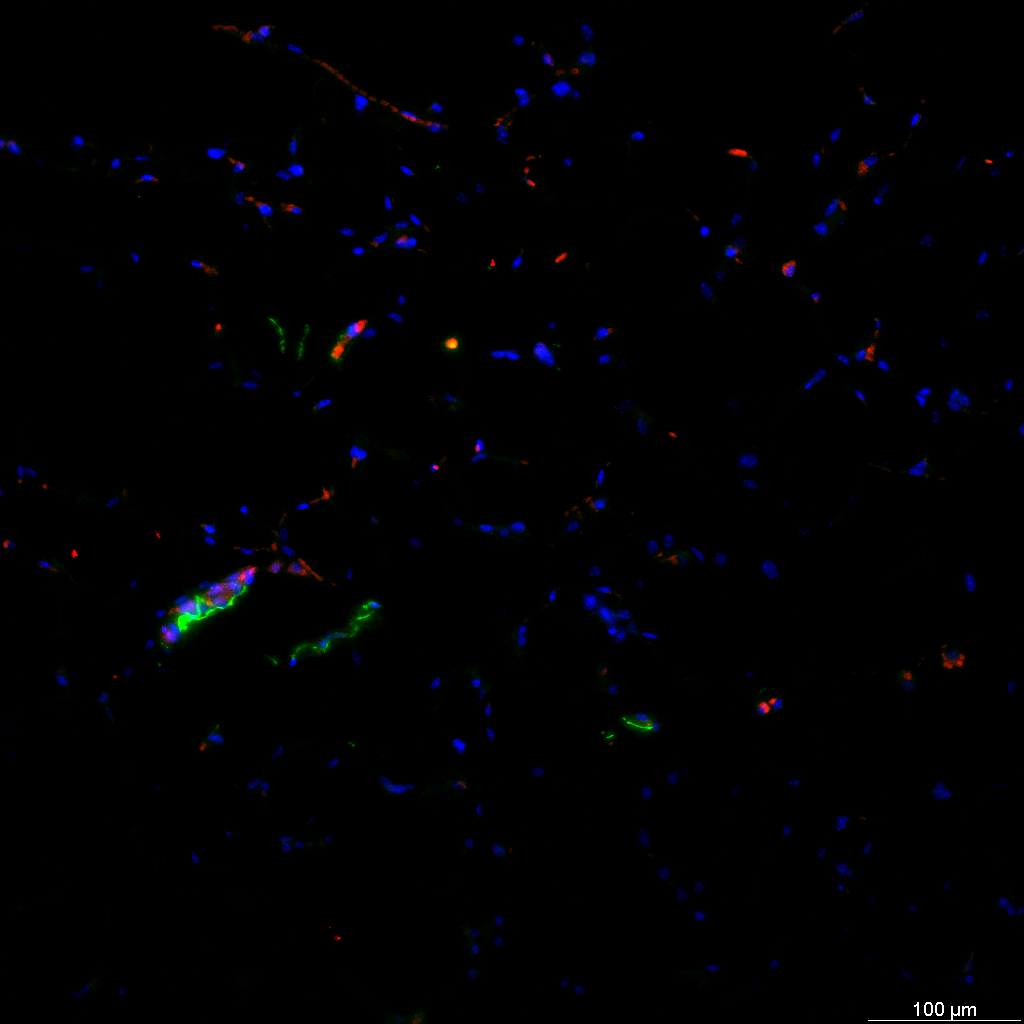

Supplement: Supplementary file 2 [file DataSheet_2.zip › Project002_Series023_Lng_LVCC_Processed001.tif]

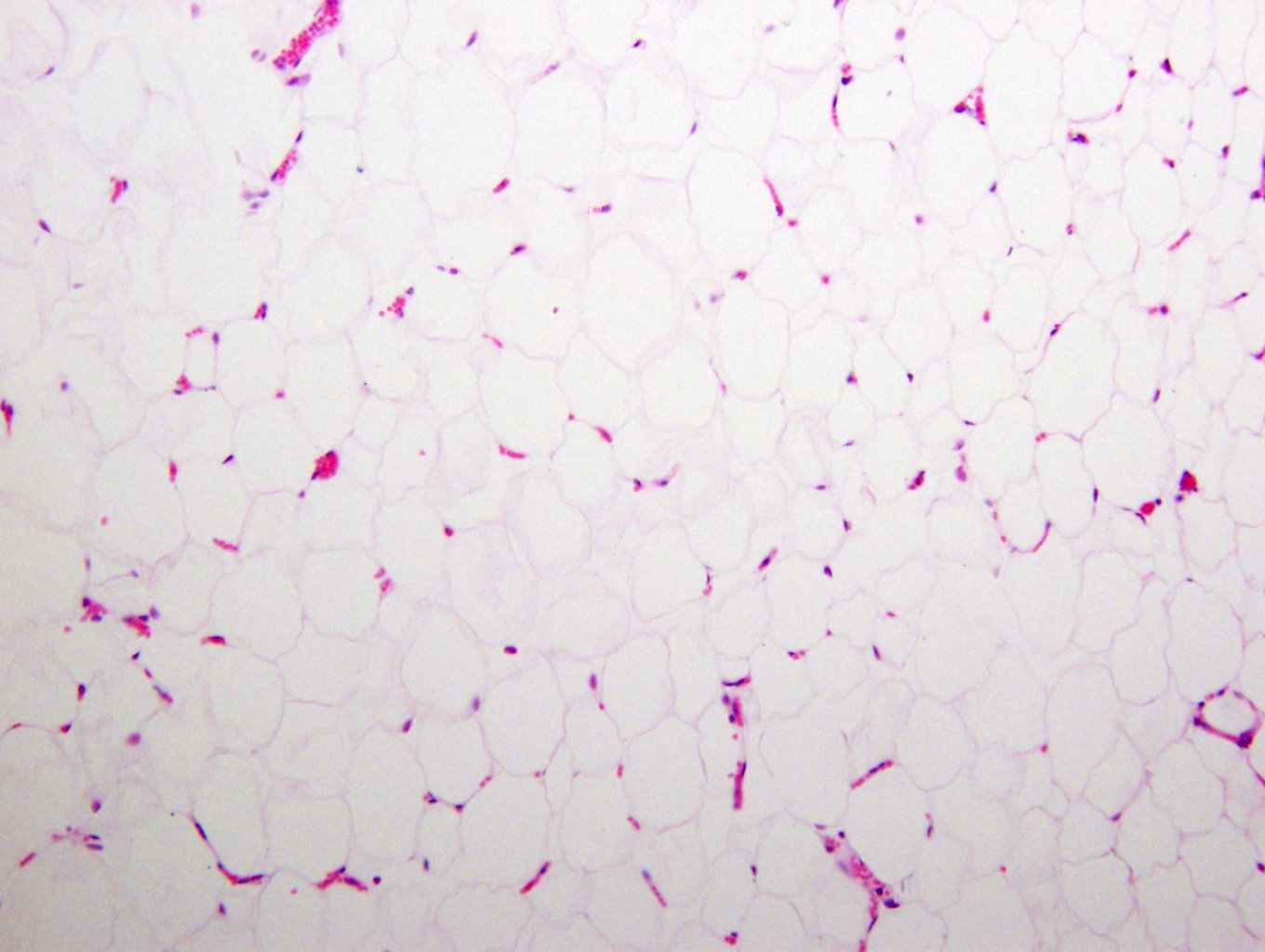

Supplement: Supplementary file 2 [file DataSheet_2.zip › ND.jpg]
